# Association Between Enteral Supplementation With High-Dose Docosahexaenoic Acid and Risk of Bronchopulmonary Dysplasia in Preterm Infants: A Systematic Review and Meta-analysis

**DOI:** 10.1001/jamanetworkopen.2023.3934

**Published:** 2023-03-21

**Authors:** Isabelle Marc, Amélie Boutin, Etienne Pronovost, Norma Maria Perez Herrera, Mireille Guillot, Frédéric Bergeron, Lynne Moore, Thomas R. Sullivan, Pascal M. Lavoie, Maria Makrides

**Affiliations:** 1 Department of Pediatrics, Centre Hospitalier Universitaire de Québec-Université Laval, Québec, Québec, Canada; 2 Library, Université Laval, Québec, Québec, Canada; 3 Department of Social and Preventive Medicine, Université Laval, Québec, Québec, Canada; 4 Women and Kids Theme, South Australian Health and Medical Research Institute, Adelaide, South Australia, Australia; 5 School of Public Health, University of Adelaide, Adelaide, South Australia, Australia; 6 Department of Pediatrics, Division of Neonatology, University of British Columbia, Vancouver, British Columbia, Canada; 7 School of Medicine, University of Adelaide, Adelaide, South Australia, Australia

## Abstract

**Importance:**

High-dose docosahexaenoic acid (DHA), a long-chain polyunsaturated fatty acid, may affect the risk of bronchopulmonary dysplasia (BPD). However, high-level summative evidence supporting such clinical association in very preterm infants is lacking.

**Objective:**

To examine the association between enteral supplementation with high-dose DHA during the neonatal period and the risk of BPD in preterm infants born at less than 29 weeks’ gestation.

**Data Sources:**

PubMed, Embase, Web of Science, Cochrane Central Register of Controlled Trials, medRxiv, and ClinicalTrials.gov were searched from inception to August 1, 2022, for eligible articles with no language restrictions.

**Study Selection:**

Randomized clinical trials (RCTs) were eligible for inclusion (1) if their interventions involved direct administration of a minimum DHA supplementation of 40 mg/kg/d or breast milk or formula feeding of at least 0.4% of total fatty acids, and (2) if they reported data on either BPD, death, BPD severity, or a combined outcome of BPD and death.

**Data Extraction and Synthesis:**

Two investigators completed independent review of titles and abstracts, full text screening, data extraction, and quality assessment using the Cochrane Risk of Bias 2.0. Risk ratios (RRs) with 95% CIs were pooled using random-effect meta-analyses.

**Main Outcomes and Measures:**

Primary outcome was BPD using trial-specific definitions, which was further stratified for RCTs that used a more stringent BPD definition based on systematic pulse oximetry assessment at 36 weeks’ postmenstrual age. Other outcomes were BPD, death, BPD severity, or combined BPD and death.

**Results:**

Among the 2760 studies screened, 4 RCTs were included, which involved 2304 infants (1223 boys [53.1%]; mean [SD] gestational age, 26.5 [1.6] weeks). Enteral supplementation with high-dose DHA was associated with neither BPD (4 studies [n = 2186 infants]; RR, 1.07 [95% CI, 0.86-1.34]; *P* = .53; *I*^2^ = 72%) nor BPD or death (4 studies [n = 2299 infants]; RR, 1.04 [95% CI, 0.91-1.18]; *P* = .59; *I*^2^ = 61%). However, an inverse association with BPD was found in RCTs that used a more stringent BPD definition (2 studies [n = 1686 infants]; RR, 1.20 [95% CI, 1.01-1.42]; *P* = .04; *I*^2^ = 48%). Additionally, DHA was inversely associated with moderate-to-severe BPD (3 studies [n = 1892 infants]; RR, 1.16 [95% CI, 1.04-1.29]; *P* = .008; *I*^2^ = 0%).

**Conclusions and Relevance:**

Results of this study showed that enteral supplementation with high-dose DHA in the neonatal period was not associated overall with BPD, but an inverse association was found in the included RCTs that used a more stringent BPD definition. These findings suggest that high-dose DHA supplementation should not be recommended to prevent BPD in very preterm infants.

## Key Points

**Question:**

Is enteral supplementation with high-dose docosahexaenoic acid (DHA) during the neonatal period associated with risk for bronchopulmonary dysplasia (BPD) at 36 weeks’ postmenstrual age in preterm infants born at less than 29 weeks’ gestation?

**Findings:**

In this systematic review and meta-analysis of 4 randomized clinical trials involving 2304 infants, enteral supplementation with high-dose DHA during the neonatal period was not associated with BPD overall but was inversely associated with BPD in trials that used a more stringent, physiological BPD definition.

**Meaning:**

Findings of this study suggest that high-dose DHA enteral supplementation should not be recommended for prevention of BPD in very preterm infants.

## Teaser

This systematic review and meta-analysis examines the association of daily omega-3 docosahexaenoic acid with bronchopulmonary dysplasia in randomized clinical trials involving preterm infants born at less than 29 weeks’ gestation.

## Introduction

Bronchopulmonary dysplasia (BPD) affects more than 40% of preterm infants who were born before 29 weeks’ gestation and presents as increased oxygen needs in the first week of life that persist beyond the neonatal period.^1^ In the long term, BPD is associated with major adverse cardiorespiratory and neurodevelopmental impairments.^2,3,4,5^ Therefore, new treatments are urgently required to reduce the incidence of BPD. Genetic factors, infections, both antenatal and postnatal inflammation, and oxidative stress events play roles in the pathogenesis of BPD.^1^ Moreover, clinical BPD definitions are continuously refined to reflect advances in noninvasive respiratory therapies in the neonatal intensive care units.^1,6^

Long-chain polyunsaturated fatty acids (LCPUFAs) and related metabolites have been hypothesized to help with repairing and healing immature lungs and thus potentially preventing BPD.^7,8,9,10,11,12^ In a post hoc analysis of the DINO (Docosahexaenoic Acid for the Improvement of Neurodevelopmental Outcome in Preterm Infants) trial, daily omega-3 docosahexaenoic acid (DHA) enteral supplementation at a high dose during the neonatal period potentially decreased the need for supplemental oxygen at 36 weeks’ postmenstrual age (PMA) in very preterm infants.^13^ However, variations in BPD definitions over time and inconsistencies in the classification of cases due to local differences in neonatal care may be associated with inaccurate estimates of the incidence of BPD across neonatal units.^1,6^ In light of these observations, more stringent BPD definitions based on a systematic assessment of the need for respiratory therapy using pulse oximetry became more widely used in more recent randomized clinical trials (RCTs) to improve the consistency of the diagnosis of BPD across neonatal units.^14,15,16,17^ Two large RCTs^18,19^ that were specifically designed to determine the effect of high-dose enteral DHA supplementation during the neonatal period on BPD outcomes in preterm infants who were born before 29 weeks’ gestation used such a systematic physiological BPD definition at 36 weeks’ PMA. Contrary to the aforementioned hypothesis, both trials reported an overall increase of BPD with the use of DHA but an inconsistent association with BPD-free survival in analysis by gestational age (GA) and mode of delivery. By contrast, early follow-ups of these trials provided some signals regarding neurodevelopment following such DHA supplementation.^20,21,22^

In light of these conflicting findings, further evidence is needed on the association of high-dose DHA enteral supplementation with BPD in very preterm infants. The aim of this systematic review and meta-analysis was to examine the association between enteral supplementation with high-dose DHA during the neonatal period and the risk of BPD in very preterm infants born at less than 29 weeks’ gestation. Additionally, we aimed to examine the differences by sex, GA at birth, mode of DHA administration, DHA source, or DHA supply with other LCPUFAs.

## Methods

The study protocol was published^23^ and registered with PROSPERO (CRD42021286705). We followed the Preferred Reporting Items for Systematic Reviews and Meta-analyses (PRISMA) reporting guideline.^24,25,26,27^

### Eligibility Criteria

Individual or cluster RCTs involving infants born at less than 29 weeks’ gestation were included. We also included studies with outcome data that were stratified and published for a subgroup of preterm infants with a GA of less than 29 weeks or an equivalent birth weight (<1250 g) if mean GA was less than 29 weeks. Conference abstracts, reviews, and case reports were not considered.

Interventions in these RCTs had to involve providing a high dose of enteral DHA omega-3 supplementation to infants either through direct administration of a minimum dose of 40 mg/kg/d or through supplementation of breast milk or formula to reach a mean percentage of DHA of at least 0.4% of total fatty acids. Although most high-dose DHA interventions target 1% of total fatty acids, a cutoff of 0.4% ensured the inclusion of any studies that used enteral supplementation over standard care. DHA could be provided alone or in conjunction with other LCPUFAs, such as arachidonic acid (AA), and supplementation had to start after birth through the neonatal period. Studies evaluating intravenous DHA supplementation were excluded since they targeted different objectives and recommendations.^23,28^ Comparison or control groups were participants who received standard care only or placebo with no or a low dose of DHA supplementation, which was defined as less than 40 mg/kg/d administered directly or less than 0.4% of total fatty acids in formula or breast milk.

To be eligible for inclusion, studies had to report data on either BPD, death, BPD severity, or a combined outcome of BPD and death. In this systematic review and meta-analysis, BPD was classified using trial-specific definitions, and analyses were stratified by the accuracy of the BPD definition based on a systematic outcome assessment by pulse oximetry at 36 weeks’ PMA.

### Data Sources and Search Strategy 

One of us (F.B., an experienced research librarian) ran searches for eligible articles with no language restrictions. Four electronic databases and 2 trial registries were searched from database inception through August 1, 2022: PubMed, Embase, Web of Science, Cochrane Central Register of Controlled Trials, medRxiv, and ClinicalTrials.gov. The search strategy used controlled vocabulary (eg, Medical Subject Heading terms) and text words and was adapted for each of the databases searched (eTables 1-6 in Supplement 1). The reference lists of identified trials, relevant systematic reviews, and protocols were also screened for any additional eligible studies (eTables 7 and 8 in Supplement 1).

Identified study references were sent to a systematic review screening software (Covidence; Veritas Health Innovation), which removed duplicates with the automation function. Two of us (I.M. and E.P.) independently screened the references to review titles and abstracts initially and then full texts later for possible inclusion according to the prespecified criteria.

### Data Extraction and Risk-of-Bias Assessment

Per the protocol,^23^ 2 of us (E.P. and N.M.P.H.) extracted data independently using a piloted data extraction form. Any discrepancies were resolved with a senior research team member (I.M.). If needed, authors of the study were contacted for additional information.

Risk of bias was assessed independently by 2 of us (E.P. and N.M.P.H.) for each of the included studies using the revised risk of bias tool (RoB, version 2.0; Cochrane Collaboration).^29^ Discrepancies were resolved with a senior research team member (A.B.).^23^

### Statistical Analysis

Meta-analysis was performed using Cochrane ReviewManager, version 5.4.1 (Cochrane Collaboration). A 2-sided *P* < .05 was considered to be statistically significant. Effect sizes were estimated as risk ratios (RRs) for treatment along with 95% CIs for each outcome. Several trials presented RRs that were adjusted for randomization stratification variables and/or data clustering or that addressed missing data using multiple imputation. As recommended, the generic inverse variance method was used to pool together the adjusted RRs, with the RRs calculated using frequencies when the effect size reported was an odds ratio or a hazard ratio.^30^

Because not all included studies presented adjusted treatment effect estimates, a sensitivity analysis was performed using the Mantel-Haenszel method to pool unadjusted RRs that were calculated from frequencies reported uniformly across studies and outcomes. We performed a random-effects meta-analysis to combine the effect estimates across trials given that the meta-analysis included fewer than 5 studies and that we anticipated a priori that the trial populations, interventions, and methods would not be sufficiently homogeneous for fixed-effects models.

Between-study heterogeneity of the effect estimates was assessed by inspecting forest plots and calculating *I*^2^ statistics. In cases of notable heterogeneity (*I*^2^ >75%), we considered its possible sources. The number of included studies was not sufficient to assess publication bias through a funnel plot.

## Results

### Trial Selection and Characteristics

**Figure 1.**
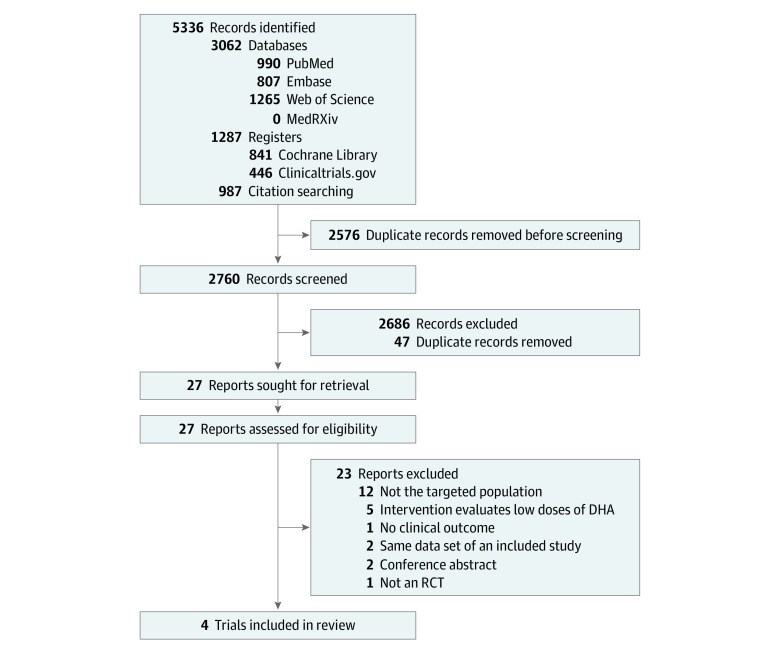
Flow Diagram of the Selection Process DHA indicates docosahexaenoic acid; RCT, randomized clinical trial.

The search yielded 5336 records, of which 2760 studies were screened. Four trials^13,18,19,31^ were included, involving 2304 very preterm infants born at less than 29 weeks’ gestation (Figure 1, Table 1; eTable 9 in Supplement 1). These infants included 1080 girls (46.9%) and 1223 boys (53.1%), with a mean (SD) GA of 26.5 (1.6) weeks. One 1 infant from the trial performed by Hellström et al^31^ was excluded from the analysis. Study and population characteristics, interventions, and BPD outcomes for the 4 included RCTs are summarized in Table 1, whereas infant characteristics are detailed by comparison or control groups and intervention groups in Table 2. Results from the DINO trial by Manley et al,^13^ involving a subgroup of infants with birth weight under 1250 g, were included in the present analyses. Aggregated data provided by the trial authors showed that the mean (SD) GA (eg, 27.2 [2.1] weeks in Manley et al^13^) was close enough to that in this study’s inclusion criterion of less than 29 weeks’ gestation (Table 2).^22^ Moreover, in the DINO trial, stratification by birth weight was performed before randomization.^13^

**Table 1.** Characteristics of Included Studies

Source^a^	Country	Population	Mode of DHA supplementation	Intervention group	Comparison or control group	Start of intervention	Duration of supplementation	Definition of BPD at 36 wks’ PMA
Collins et al,^18^ 2017	Australia	1273 infants with <29 wks’ GA	Direct enteral supplementation to the infant	Tuna oil: DHA 60 mg/kg/d	Soy oil	First enteral feeding	Until 36 wks’ PMA or discharge home	Physiological BPD (room air challenge): need for supplemental oxygen and/or respiratory support^b^
Hellström et al,^31^ 2021	Sweden	207 infants with <28 wks’ GA	Direct enteral supplementation to the infant	Blend of algal, fungal, and sunflower oil: DHA 50 mg/kg/d and AA 100 mg/kg/d	None	Birth	Until 40 wks’ PMA	Need for supplemental oxygen
Manley et al,^13^ 2011 (subgroup of Makrides et al,^22^ 2009)	Australia	296 infants with <1250 g birth weight	Enriched breast milk through maternal supplementation or enriched formula if breast milk not available	Tuna oil: DHA 900 mg/d (1% breast milk concentration) or formula enriched with 1% DHA	Soy oil	First enteral feeding	Until expected date of delivery	Need for supplemental oxygen
Marc et al,^19^ 2020	Canada	528 infants with <29 wks’ GA	Enriched breast milk through maternal supplementation	Algal oil: DHA 1200 mg/d (1% breast milk concentration)	Soy and corn oil	≤72 h after delivery	Until 36 wks’ PMA	Physiological BPD (room air challenge): need for supplemental oxygen and/or respiratory support^b^

Abbreviations: AA, arachidonic acid; BPD, bronchopulmonary dysplasia; DHA, docosahexaenoic acid; GA, gestational age; PMA, postmenstrual age.

^a^ All are individual randomized clinical trials.

^b^ Based on oximetry.^32^

**Table 2.** Reported Infant Characteristics in Included Studies

Characteristic	Infants, No. (%)							
	Collins et al,^18^ 2017		Hellström et al,^31^ 2021		Manley et al,^13^ 2011		Marc et al,^19^ 2020	
	Intervention group	Comparison or control group	Intervention group	Comparison or control group	Intervention group	Comparison or control group	Intervention group	Comparison or control group
No. of infants	631	642	101	105	147	149	273	255
Sex								
Female	293 (46.4)	300 (46.7)	43 (42.6)	46 (43.8)	70 (47.6)	75 (50.3)	135 (49.5)	118 (46.3)
Male	338 (53.6)	342 (53.3)	58 (57.4)	59 (56.2)	77 (52.4)	74 (49.7)	138 (50.5)	137 (53.7)
Gestational age, mean (SD), wk	26.7 (1.5)	26.7 (1.5)	25.5 (1.5)	25.5 (1.4)	27.2 (2.0)	27.2 (2.2)	26.7 (1.5)	26.4 (1.6)
Gestational age <27 wk	322 (51.0)	317 (49.4)	78 (77.2)	86 (81.9)	52 (35.4)	53 (35.6)	142 (52.0)	147 (57.6)
Birth weight, mean (SD), g	913 (236)	924 (239)	797 (197)	777 (197)	916 (199)	922 (215)	898 (242)	892 (237)
Exposure to antenatal corticosteroids	586 (92.9)	592 (92.4) [n = 641]	NA	NA	137 (93.2)	138 (92.6)	256 (94.5) [n = 271]	235 (93.3) [n = 252]
Cesarean birth	373 (59.1)	373 (58.1)	61 (60.4)	65 (61.9)	113 (76.9)	105 (70.5)	199 (73.4) [n = 271]	145 (57.5) [n = 252]
Exposure to IV DHA	27 (4.3) [n = 628]	32 (5.0) [n = 641]	NA	NA	IV DHA not used	IV DHA not used	144 (52.8)	128 (50.2)
Reported intervention fidelity, mean (SD), % of expected dose received	90.0 [n = NA]^a^	90.0 [n = NA]^a^	88.9 (20.5) [n = NA]	No intervention	86.7 [n = 97]^a^	88.3 [n = 91]^a^	73.3 (28.7) [n = 248]	74.2 (27.0) [n = 224]
DHA level in whole blood, mean (SD)^b^	3.9 (0.7	2.5 (0.6	No blood sample	No blood sample	No blood sample	No blood sample	No blood sample	No blood sample
DHA level in serum, mean (95% CI), molar ratio	No serum sample	No serum sample	2.20 (2.11-2.29)	2.07 (2.00-2.15)	No serum sample	No serum sample	No serum sample	No serum sample
DHA level in maternal breast milk, mean (SD)^b^	No breast milk sample	No breast milk sample	No breast milk sample	No breast milk sample	0.85 (0.39)	0.25 (0.13)	0.95 (0.44)	0.34 (0.20)
DHA level in formula milk, mean (SD)^b^	No formula milk used	No formula milk used	No formula milk used	No formula milk used	1.11 (0.29)	0.42 (0.05)	NA	NA

Abbreviations: DHA, docosahexaenoic acid; IV, intravenous; NA, not available.

^a^ SD was not available.

^b^ DHA level was the % of total fatty acids.

In terms of interventions, high-dose DHA supplementation in these RCTs was either provided directly to the infants^18,31^ or through DHA-enriched breast milk or formula.^13,19^ The DHA sources were fish oil^13,18^; algal oil^19^; or a mix of algal, fungal, and sunflower oils combined with AA supplementation.^31^ Comparison or control groups received either a placebo with no additional DHA (ie, soy oil; soy and corn oil)^13,18,19^ or no supplement^31^ (Table 1).

In terms of outcome definitions, the RCTs differed by the criteria they used to define BPD (Table 1). In 2 trials, BPD was defined based on the need for supplemental oxygen at 36 weeks’ PMA, but no systematic oximetry assessment was performed.^13,31^ In the other 2 trials, BPD was defined as either physiological BPD^18^ or BPD-free survival^19^ based on the need for supplemental oxygen and/or respiratory support, which was systematically assessed at 36 weeks’ PMA by pulse oximetry.^32^

Two trials^18,19^ further classified BPD severity according to the National Institute of Child Health and Human Development.^33^ Hellström et al^31^ reported rates for mild, moderate, and severe BPD but without references on definitions. No data on BPD severity were reported in the DINO trial.^13^ All RCTs were assessed as having low risk of bias, except 1 trial^31^ that was classified as having potential bias due to the unmasked clinicians in charge of respiratory care management and the assessors of the BPD outcome (eFigure 1 in Supplement 1).

### Quantitative Data Synthesis of BPD Incidence and Other Outcomes

**Figure 2.**
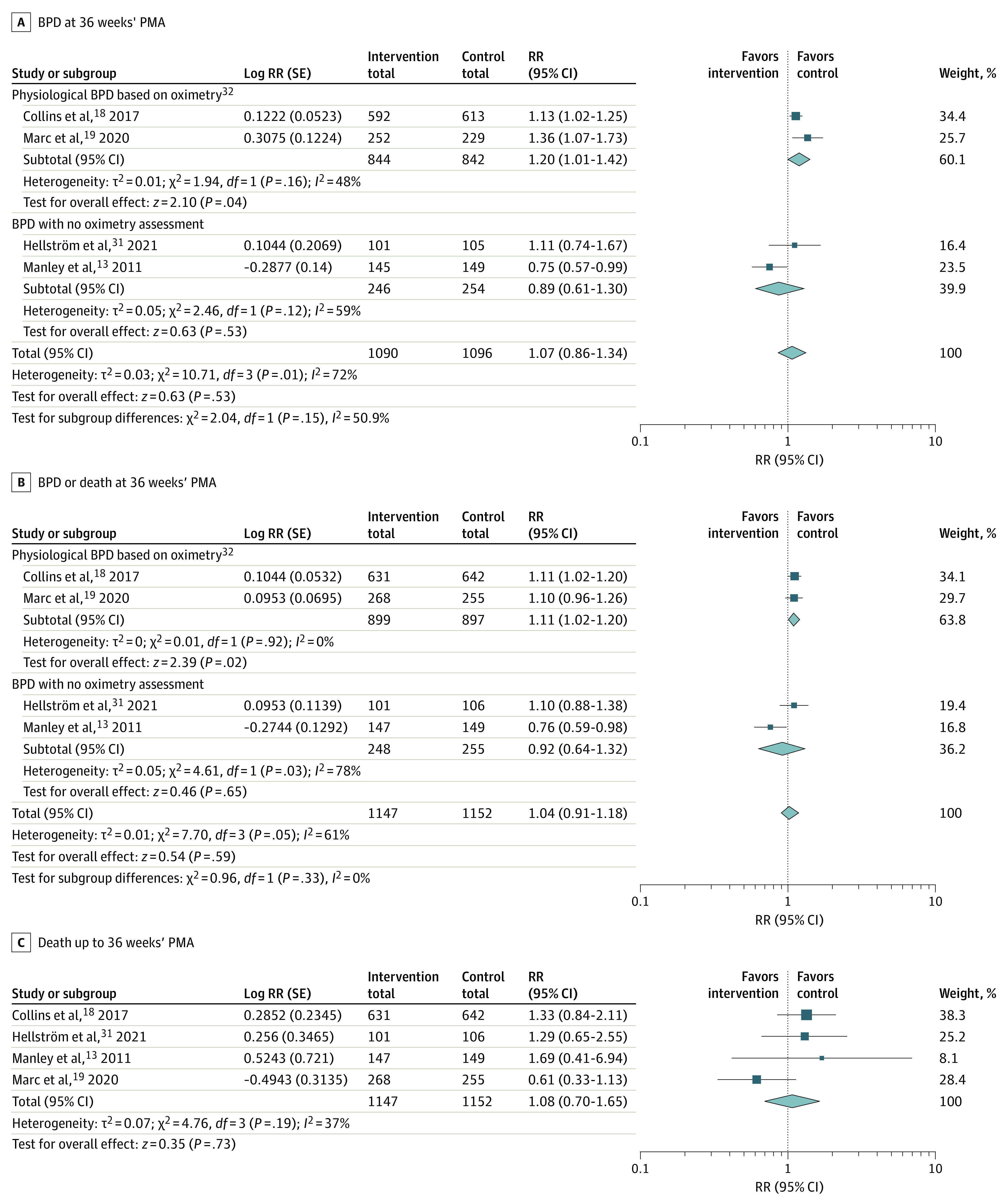
Meta-analysis Comparing High-Dose Docosahexaenoic Acid Supplementation With a Control on Bronchopulmonary Dysplasia (BPD) and Death In panels B and C, death was analyzed up to 36 weeks' postmenstrual age (PMA) except in Hellström et al,^31^ where death was analyzed up to 40 weeks' PMA. PMA indicates postmenstrual age; RR, risk ratio.

In trials using trial-specific BPD definitions, providing enteral supplementation with high-dose DHA in the neonatal period to very preterm infants born at less than 29 weeks’ gestation was not associated with either BPD (4 trials^13,18,19,31^ [n = 2186 infants]; RR, 1.07 [95% CI, 0.86-1.34]; *P* = .53; *I*^2^ = 72%) (Figure 2A) or BPD or death (4 trials^13,18,19,31^ [n = 2299 infants]; RR, 1.04 [95% CI, 0.91-1.18]; *P* = .59; *I*^2^ = 61%) (Figure 2B) at 36 weeks’ PMA. Results that were stratified for trials reporting on BPD based on a systematic oximetry at 36 weeks’ PMA (2 trials^18,19^ [n = 1801 infants]) are presented in Figure 2A. Compared with the control group, in the intervention group enteral supplementation with high-dose DHA was associated with a significantly increased risk of BPD at 36 weeks’ PMA (2 trials^18,19^ [n = 1686 infants]; RR, 1.20 [95% CI, 1.01-1.42]; *P* = .04; *I*^2^ = 48%). Similarly, the risk for physiological BPD or death at 36 weeks’ PMA was also associated with a high dose of DHA supplementation (2 trials^18,19^ [n = 1796 infants]; RR, 1.11 [95% CI, 1.02-1.20]; *P* = .02; *I*^2^ = 0%) (Figure 2B). No association of DHA supplementation with mortality was detected (4 trials^13,18,19,31^ [n = 2299 infants]; RR, 1.08 [95% CI, 0.70-1.65]; *P* = .73; *I*^2^ = 37%) (Figure 2C).

**Figure 3.**
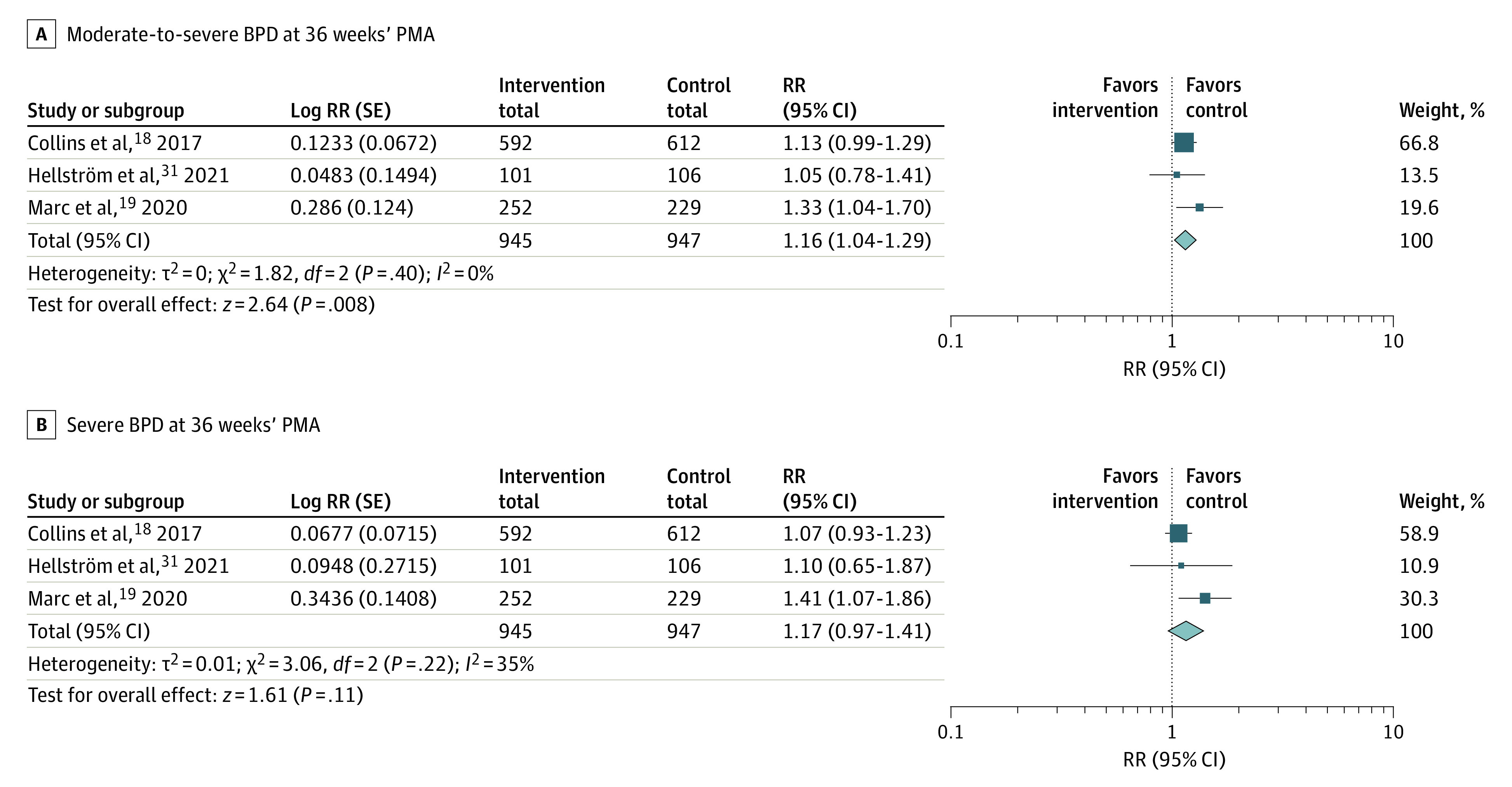
Meta-analysis Comparing High-Dose Docosahexaenoic Acid Supplementation With a Control on Bronchopulmonary Dysplasia (BPD) Severity The BPD severity was classified according to criteria from the National Institute of Child Health and Human Development^33^ in Collins et al^18^ and Marc et al.^19^ A definition was not specified in Hellström et al.^31^ PMA indicates postmenstrual age; RR, risk ratio.

In trials using a severity-based BPD definition, the risk of moderate-to-severe BPD at 36 weeks’ PMA was also associated with the intervention group vs the comparison or control group (3 trials^18,19,31^ [n = 1892 infants]; RR, 1.16 [95% CI, 1.04-1.29]; *P* = .008; *I*^2^ = 0%) (Figure 3A). Although not significant, the risk for severe BPD was associated with the intervention group (3 trials^18,19,31^ [n = 1892 infants]; RR, 1.17 [95% CI, 0.97-1.41]; *P* = .11; *I*^2^ = 35%) (Figure 3B).

### Sensitivity and Subgroup Analyses

Sensitivity meta-analyses performed by calculating RRs from frequencies for all included studies showed results similar to those of the main analysis, which was based on RRs calculated from adjusted RRs (eFigure 2 in Supplement 1. Interactions of sex and GA for the associations between DHA and BPD were not significant (eFigures 3 and 4 in Supplement 1). No associations were observed by mode of DHA administration, source of DHA, and whether DHA was supplemented with AA, although the findings of trials that used direct mode of administration^18,31^ or algal oil^19,31^ suggested that DHA was associated with increased risk for BPD (eFigure 5 in Supplement 1).

## Discussion

In this systematic review and meta-analysis of 4 RCTs involving 2304 infants born at less than 29 weeks’ gestation, enteral supplementation with high-dose DHA in the neonatal period was not associated with BPD and BPD or death overall in trials using trial-specific BPD definitions,^13,18,19,31^ although high heterogeneity limited the interpretation of these results. However, an inverse association with BPD and BPD or death was found for trials^18,19^ using more stringent BPD definitions based on a systematic assessment by pulse oximetry at 36 weeks’ PMA. Moreover, enteral supplementation with high-dose DHA was associated with increased risks of moderate-to-severe BPD and severe BPD. The lack of association between DHA and death suggested that the increased risk of moderate-to-severe BPD associated with DHA was not due to increased survival in these infants. No difference in the association between DHA supplementation and BPD was found according to sex, GA, mode of DHA administration, source of DHA, or whether DHA was supplemented with AA.

These findings updated those of previous systematic reviews^34,35,36^ that suggested no association with BPD or protective benefits of DHA supplementation against BPD in infants with GA of 32 weeks’ or less. However, the inclusion criteria varied between systematic reviews and were associated with heterogeneity in targeted populations, intervention doses, as well as BPD assessments and cointerventions (eg, respiratory care management).^34,35,36^ Moreover, recent trials were also inconsistently included in these systematic reviews.^18,19,31^ Unlike previous systematic reviews, the present study focused on the outcome of high-dose DHA supplementation in infants born at less than 29 weeks’ gestation and at greater risk of BPD.^1^

Variations in BPD rates have been reported between units in part due to infants not being systematically assessed using pulse oximetry at 36 weeks’ PMA.^1,6^ This issue was addressed in 2 large and more recent RCTs^18,19^ that were specifically designed to determine the effect of DHA on BPD and were conducted subsequent to the DINO trial.^13^ Alternatively, the use of a BPD definition, classified on its severity according to the mode of respiratory support regardless of the need for supplemental oxygen, may be a better outcome associated with early childhood morbidities in modern neonatal respiratory care.^37^ Results of this systematic review and meta-analysis did not detect an association between DHA and severe BPD, yet the inverse association of DHA with moderate-to-severe BPD warrants investigation to understand how to balance the potential adverse implications of DHA for BPD with the potential benefits of DHA for neurodevelopment.^20,21,22^

Two trials^19,31^ reported an increased risk for BPD after DHA administration in more mature babies, resulting in a higher effect size in this category. However, stratification according to GA was inconclusive, with no subgroup differences found and high evidence of heterogeneity limited the interpretation of results. Similarly, no difference was detected in whether DHA was administered directly or through maternal breast milk, although maternal supplementation may have played a role in the gradual intake of DHA in a readily bioavailable form after mammary gland processing.^38,39^ However, it is important to point out that subgroup analyses in strata are exploratory and should be considered with caution.

### Biological and Clinical Plausibility

Given the lack of association or potentially harmful associations between DHA and BPD in infants born at less than 29 weeks’ gestation, the results of this systematic review and meta-analysis can be pragmatically interpreted as such: supplementing a high dose of enteral DHA to very preterm infants cannot be recommended for the prevention of BPD. However, more studies are needed to understand the mechanisms involved, as DHA was expected to play a role in the reduction of oxidative stress and inflammation underlying the pathogenesis of BPD.

This study did not allow the comparison of the association of DHA alone with the association of DHA in combination with AA. The trial by Hellström et al^31^ was the only one that evaluated the effect of combined DHA and AA supplementation on BPD and showed there was no association with BPD; this finding was similar to that of a recent secondary analysis^40^ of another RCT published after the completion of the present study. By contrast, data suggested a benefit of DHA alone for inflammation in preterm infants^7,8,9^ and for lung architecture in animals,^10,11^ with observational studies also supporting a reduced risk of chronic lung disease with higher postnatal levels of DHA in very preterm infants.^12^ High-dose DHA enteral supplementation should not have substantially modified the AA intake.^41,42^ However, it is plausible that imbalance in the AA to DHA ratio may be associated with the impaired mechanisms involved in the active resolution of inflammation, potentially resulting in increased BPD when DHA is supplemented in the absence of sufficient AA.^43,44,45^

In this systematic review and meta-analysis, lack of evidence and heterogeneity across RCTs that used DHA alone limited the interpretation of the results. Large, adequately powered and designed trials are needed to assess the value of adding AA to high-dose DHA supplementation.

### Clinical and Research Recommendations

Based on the current available evidence on BPD, high-dose enteral DHA supplementation during the neonatal period cannot be recommended for prevention of BPD in very preterm infants. Additionally, the use of DHA supplements by mothers who provide their own breast milk to their very preterm infants cannot be recommended for prevention of BPD.

To increase confidence in the study results, a meta-analysis of individual participant data should be performed using a harmonized BPD definition that better reflects neonatal care in current practice and that estimates serious respiratory and neurodevelopmental morbidities in early childhood.^37^ Beyond the clinical significance of the association of DHA with short-term neonatal outcomes, long-term follow-up of these cohorts is required because the potential benefits for long-term neurodevelopment and cardiorespiratory health would need to be balanced against the potential increased risk of BPD. In addition to these research recommendations, documenting the dose-response associations between lipids metabolism and outcomes will contribute to overall health improvement in very preterm infants by implementing better practices in neonatal nutrition.

### Strengths and Limitations

Although only 4 studies were included in this systematic review and meta-analysis, 2304 infants were involved in these multicenter RCTs. However, all 4 RCTs were conducted in industrialized countries with access to level 3 neonatal care units, which could limit the generalizability of the results to infants who may not benefit from the latest sophisticated neonatal care. Although the main analyses using trial-specific BPD definitions were justified, further stratification by more stringent BPD classification preserves the prominence of the predefined BPD outcome in the study protocol.^23^

## Conclusions

In this systematic review and meta-analysis of 4 RCTs, enteral supplementation with high-dose DHA in the neonatal period was not associated overall with BPD in infants born at less than 29 weeks’ gestation, but an inverse association was found for the trials that used a more stringent BPD definition that was based on use of pulse oximetry. These results suggest that high-dose DHA enteral supplementation should not be recommended for prevention of BPD in very preterm infants. Further research is needed to understand the association between high-dose DHA supplementation and BPD as well as how this association affects other short-term and long-term outcomes.

## Notes

Supplement 1. **eTable 1.** PubMed Search Strategy and Results **eTable 2.** Embase Search Strategy and Results **eTable 3.** Cochrane Library Search Strategy and Results **eTable 4.** Web of Science Search Strategy and Results **eTable 5.** MedRXiv Search Strategy and Results **eTable 6.** Clinicaltrials.gov Search Strategy and Results **eTable 7.** Citation Searching Details and Results **eTable 8.** Status of Protocols for Citation Searching **eTable 9.** Reasons for Exclusion at Full-Text Assessment **eFigure 1.** Risk of Bias Assessment of Included Trials **eFigure 2.** Meta-analysis Pooling Risk Ratio From Reported Frequencies on BPD Outcomes **eFigure 3.** Meta-analysis Results on BPD Outcome Stratified by Sex **eFigure 4.** Meta-analysis Results on BPD Outcome Stratified by Gestational Age **eFigure 5.** Meta-analysis Results on BPD Outcome Stratified by Mode of Administration, Source of DHA and Type of Supplementation
eReferences

Supplement 2.
Data Sharing Statement
