# Supplementary material for: Association Between Enteral Supplementation With High-Dose Docosahexaenoic Acid and Risk of Bronchopulmonary Dysplasia in Preterm Infants: A Systematic Review and Meta-analysis
Source: JAMA Netw Open. 2023 Mar 21;6(3):e233934. doi: 10.1001/jamanetworkopen.2023.3934 (PMC10031388; doi:10.1001/jamanetworkopen.2023.3934)
Supplement: Supplement 1. — eTable 1. PubMed Search Strategy and Results eTable 2. Embase Search Strategy and Results eTable 3. Cochrane Library Search Strategy and Results eTable 4. Web of Science Search Strategy and Results eTable 5. MedRXiv Search Strategy and Results eTable 6. Clinicaltrials.gov Search Strategy and Results eTable 7. Citation Searching Details and Results eTable 8. Status of Protocols for Citation Searching eTable 9. Reasons for Exclusion at Full-Text Assessment eFigure 1. Risk of Bias Assessment of Included Trials eFigure 2. Meta-analysis Pooling Risk Ratio From Reported Frequencies on BPD Outcomes eFigure 3. Meta-analysis Results on BPD Outcome Stratified by Sex eFigure 4. Meta-analysis Results on BPD Outcome Stratified by Gestational Age eFigure 5. Meta-analysis Results on BPD Outcome Stratified by Mode of Administration, Source of DHA and Type of Supplementation eReferences [file jamanetwopen-e233934-s001.pdf]

Supplemental Online Content

Marc I, Boutin A, Pronovost E, et al. Association between enteral supplementation with high-dose docosahexaenoic acid and risk of bronchopulmonary dysplasia in preterm infants: a systematic review and meta-analysis. *JAMA Netw Open*. 2023;6(3):e233934. doi:10.1001/jamanetworkopen.2023.3934

eTable 1. PubMed Search Strategy and Results

eTable 2. Embase Search Strategy and Results

eTable 3. Cochrane Library Search Strategy and Results

eTable 4. Web of Science Search Strategy and Results

eTable 5. MedRXiv Search Strategy and Results

eTable 6. Clinicaltrials.gov Search Strategy and Results

eTable 7. Citation Searching Details and Results

eTable 8. Status of Protocols for Citation Searching

eTable 9. Reasons for Exclusion at Full-Text Assessment

eFigure 1. Risk of Bias Assessment of Included Trials

eFigure 2. Meta-analysis Pooling Risk Ratio From Reported Frequencies on BPD Outcomes

eFigure 3. Meta-analysis Results on BPD Outcome Stratified by Sex

eFigure 4. Meta-analysis Results on BPD Outcome Stratified by Gestational Age

eFigure 5. Meta-analysis Results on BPD Outcome Stratified by Mode of Administration, Source of DHA and Type of Supplementation

eReferences

This supplementary material has been provided by the authors to give readers additional information about their work.

© 2023 Marc I et al. *JAMA Network Open.*

eTable 1. *PubMed* search strategy and results

#	Search strategy	Results
**1**	"Fatty Acids, Omega-3"[Mesh:NoExp] OR "Docosahexaenoic Acids"[Mesh] OR "Fish Oils"[Mesh:NoExp] OR "Fatty Acids, Essential"[Mesh:noexp]	31,433
**2**	Omega 3[TIAB] OR n 3 fatty acid[TIAB] OR n 3 Oil[TIAB] OR n3 Oil[TIAB] OR Polyunsaturated Fatty Acid*[TIAB] OR polyunsaturated FA[TIAB] OR Docosahexaenoic Acid*[TIAB] OR alga* oil[TIAB] OR marine oil[TIAB] OR fish oil[TIAB] OR essential fatty acid*[TIAB] OR DHA[TIAB]	61,759
**3**	#1 OR #2	67,434
**4**	"Infant, Premature"[Mesh] OR "Infant, Low Birth Weight"[Mesh] OR "Premature Birth"[Mesh] OR "Infant, Newborn"[Mesh]	645,200
**5**	Premature Infant*[TIAB] OR preterm infant*[TIAB] OR low birth weight[TIAB] OR preterm birth*[TIAB] OR Premature Birth*[TIAB] OR newborn*[TIAB] OR neonate*[TIAB]	334,113
**6**	#4 OR #5	760,745
**7**	((randomized controlled trial[pt]) OR (controlled clinical trial[pt]) OR (randomized[tiab] OR randomised[tiab]) OR (placebo[tiab]) OR (drug therapy[sh]) OR (randomly[tiab]) OR (trial[tiab]) OR (groups[tiab]))	5,254,468
**8**	(animals[MeSH] NOT humans[MeSH])	4,907,993
**9**	(#3 AND #6 AND #7) NOT #8	949
**10**	#9 AND 2021/11/02:2022/08/01[edat]	41
**11**	**Total PubMed results**	990

© 2023 Marc I et al. *JAMA Network Open.*

eTable 2. *Embase* search strategy and results

#	Search strategy	Results
**1**	'omega 3 fatty acid'/de OR 'docosahexaenoic acid'/de OR 'fish oil'/de OR 'essential fatty acid'/de	63,631
**2**	"Omega 3":ti,ab,kw OR "n 3 fatty acid":ti,ab,kw OR "n 3 Oil":ti,ab,kw OR "n3 Oil":ti,ab,kw OR "Polyunsaturated Fatty Acid$":ti,ab,kw OR "Polyunsaturated FA":ti,ab,kw OR "Docosahexaenoic Acid$":ti,ab,kw OR "alga* oil":ti,ab,kw OR "marine oil":ti,ab,kw OR "fish oil":ti,ab,kw OR "essential fatty acid$":ti,ab,kw OR DHA:ti,ab,kw	74,163
**3**	#1 OR #2	94,604
**4**	'prematurity'/de OR 'low birth weight'/exp OR 'very low birth weight'/exp OR 'newborn'/de	723,734
**5**	"Premature Infant$":ti,ab,kw OR "preterm infant$":ti,ab,kw OR "low birth weight":ti,ab,kw OR "preterm birth$":ti,ab,kw OR "Premature Birth$":ti,ab,kw OR newborn$:ti,ab,kw OR neonate$:ti,ab,kw	424,026
**6**	#4 OR #5	845,949
**7**	(("Randomized controlled trial"/de) OR ("Controlled clinical trial"/de) OR (random*:ti,ab) OR (randomization/de) OR ("intermethod comparison"/de) OR (placebo:ti,ab) OR (compare OR compared OR comparison):ti OR ((evaluated OR evaluate OR evaluating OR assessed OR assess) AND (compare OR compared OR comparing OR comparison)):ti OR (open NEAR/1 label):ti,ab OR ((double OR single OR doubly OR singly) NEAR/1 (blind OR blinded OR blindly)):ti,ab OR ("double blind procedure"/de) OR ("parallel group*":ti,ab) OR (crossover OR "cross over"):ti,ab OR ((assign* OR match OR matched OR allocation) NEAR/5 (alternate OR group* OR intervention* OR patient* OR subject* OR participant*)):ti,ab OR (assigned OR allocated):ti,ab OR (controlled NEAR/7 (study OR design OR trial)):ti,ab OR (volunteer OR volunteers):ti,ab OR ("human experiment"/de) OR (trial:ti))	3,923,157
**8**	((random* NEAR/1 sampl* NEAR/7 ("cross section*" or questionnaire* or survey* or database*)):ti,ab NOT ("comparative study"/de OR "controlled study"/de OR "randomi$ed controlled":ti,ab OR "randomly assigned":ti,ab) OR ("Cross-sectional study"/de NOT ("randomized controlled trial"/de OR "controlled clinical study"/de OR "controlled study"/de OR "randomi$ed controlled":ti,ab OR "control group*":ti,ab)) OR (((case NEAR/1 control*) AND random*) NOT "randomi$ed controlled"):ti,ab OR ("Systematic review" NOT (trial OR study)):ti OR (nonrandom* NOT random*):ti,ab OR ("Random field*":ti,ab) OR ("random cluster" NEAR/3 sampl*):ti,ab OR (review:ab AND review/de) NOT trial:ti OR ("we searched":ab AND (review:ti OR review/de)) OR ("update review":ab) OR (databases NEAR/4 searched):ab OR ((rat OR rats OR mouse OR mice OR swine OR porcine OR murine OR sheep OR lambs OR pigs OR piglets OR rabbit OR rabbits OR cat OR cats OR dog OR dogs OR cattle OR bovine OR monkey OR monkeys OR trout OR marmoset*):ti AND "animal experiment"/de) OR ("Animal experiment"/de NOT ("human experiment"/de OR human/de)))	3,751,550
**9**	(#3 AND #6 AND #7) NOT #8	758
**10**	#9 AND [02-11-2021]/sd	49
**11**	**Total Embase results**	807

© 2023 Marc I et al. *JAMA Network Open.*

eTable 3. *Cochrane Library* search strategy and results

#	Search strategy	Results
**1**	[mh ^"Fatty Acids, Omega-3"] OR [mh "Docosahexaenoic Acids"] OR [mh "Fish Oils"] OR [mh ^"Fatty Acids, Essential"]	3,747
**2**	"Omega 3":ti,ab,kw OR "n 3 fatty acid":ti,ab,kw OR "n 3 Oil":ti,ab,kw OR "n3 Oil":ti,ab,kw OR "Polyunsaturated Fatty Acid":ti,ab,kw OR "Polyunsaturated Fatty Acids":ti,ab,kw OR "Polyunsaturated FA":ti,ab,kw OR "Docosahexaenoic Acid":ti,ab,kw OR "Docosahexaenoic Acids":ti,ab,kw OR (alga* near/1 oil):ti,ab,kw OR "marine oil":ti,ab,kw OR "fish oil":ti,ab,kw OR "essential fatty acid":ti,ab,kw OR "essential fatty acids":ti,ab,kw OR DHA:ti,ab,kw	10,907
**3**	#1 OR #2	11,154
**4**	[mh "Infant, Premature"] OR [mh "Infant, Low Birth Weight"] OR [mh "Premature Birth"] OR [mh "Infant, Newborn"]	18,086
**5**	((premature OR preterm) NEAR/1 (Infant* OR birth*)):ti,ab,kw OR "low birth weight":ti,ab,kw OR newborn*:ti,ab,kw OR neonate*:ti,ab,kw	39,945
**6**	#4 OR #5	39,958
**7**	#3 AND #6	810
**8**	Trials results only	798
**9**	Date added to CENTRAL trials database limit 02/11/2021 to 01/08/2022	43
**10**	**Total Cochrane Library results**	841

© 2023 Marc I et al. *JAMA Network Open.*

eTable 4. *Web of Science* search strategy and results

#	Search strategy	Results
**1**	TS=("Omega 3" OR "n 3 fatty acid" OR "n 3 Oil" OR "n3 Oil" OR "Polyunsaturated Fatty Acid$" OR "Polyunsaturated FA" OR "Docosahexaenoic Acid$" OR "alga* oil" OR "marine oil" OR "fish oil" OR "essential fatty acid*" OR DHA)	96,862
**2**	TS=(((premature OR preterm) NEAR/1 (Infant$ OR birth$)) OR "low birth weight" OR newborn$ OR neonate$)	331,476
**3**	TS=(randomized OR randomised OR placebo OR randomly OR trial OR groups OR "drug therapy")	7,238,171
**4**	#1 AND #2 AND #3	1,219
**5**	Publication date database limit 2021-11-02 to 2022-08-01	46
**6**	**Total Web of Science results**	1265

© 2023 Marc I et al. *JAMA Network Open.*

eTable 5. *MedRXiv* search strategy and results

#	Search strategy	Results
**1**	("omega 3" OR "n 3 fatty acid" OR "n 3 Oil" OR "n3 Oil" OR "polyunsaturated Fatty Acid" OR "polyunsaturated Fatty Acids" OR "polyunsaturated FA" OR "docosahexaenoic Acid" OR "docosahexaenoic Acids" OR "alga oil" OR "marine oil" OR "fish oil" OR "essential fatty acid" OR "essential fatty acids" OR DHA) AND ("Premature Infant" OR "Premature Infants" OR "preterm infant" OR "preterm infants" OR "low birth weight" OR "preterm birth" OR "preterm births" OR "premature Birth" OR "Premature Birth" OR newborn OR newborns OR neonate OR newborns)	0
**2**	Publication date database limit 2021-11-02 to 2022-08-01	0
**3**	**Total MedRxiv results**	0

© 2023 Marc I et al. *JAMA Network Open.*

eTable 6. *Clinicaltrials.gov* search strategy and results

#	Search strategy[a]	Results
**1**	("omega 3" OR "n 3 fatty acid" OR "n 3 Oil" OR "n3 Oil" OR "polyunsaturated Fatty Acid" OR "polyunsaturated Fatty Acids" OR "polyunsaturated FA" OR "docosahexaenoic Acid") AND ("Premature Infant" OR "Premature Infants" OR "preterm infant" OR "preterm infants" OR "low birth weight" OR "preterm birth" OR "preterm births" OR "premature Birth" OR "Premature Birth" OR newborn OR newborns OR neonate OR newborns)	97
**2**	("docosahexaenoic Acids" OR "alga oil" OR "marine oil" OR "fish oil" OR "essential fatty acid" OR "essential fatty acids" OR DHA) AND ("Premature Infant" OR "Premature Infants" OR "preterm infant" OR "preterm infants" OR "low birth weight" OR "preterm birth" OR "preterm births" OR "premature Birth" OR "Premature Birth" OR newborn OR newborns OR neonate OR newborns)	117
**3**	#1 OR #2	214
**New search on 2022-08-01**		
**1**	("omega 3" OR "n 3 fatty acid" OR "n 3 Oil" OR "n3 Oil" OR "polyunsaturated Fatty Acid" OR "polyunsaturated Fatty Acids" OR "polyunsaturated FA" OR "docosahexaenoic Acid") AND ("Premature Infant" OR "Premature Infants" OR "preterm infant" OR "preterm infants" OR "low birth weight" OR "preterm birth" OR "preterm births" OR "premature Birth" OR "Premature Birth" OR newborn OR newborns OR neonate OR newborns)	105
**2**	("docosahexaenoic Acids" OR "alga oil" OR "marine oil" OR "fish oil" OR "essential fatty acid" OR "essential fatty acids" OR DHA) AND ("Premature Infant" OR "Premature Infants" OR "preterm infant" OR "preterm infants" OR "low birth weight" OR "preterm birth" OR "preterm births" OR "premature Birth" OR "Premature Birth" OR newborn OR newborns OR neonate OR newborns)	127
**3**	#1 OR #2	232

[a]Search was executed in the "Intervention/treatment" and "Other terms" fields.

© 2023 Marc I et al. *JAMA Network Open.*

eTable 7. Citation searching details and results

Seminal papers	Backward citations	Forward citations	Tool/method use	Results
Reviews known by the research team (28)	X		Web of Science	765
Included studies (4)	X	X	Web of Science, iCite and CitationChaser	161
Protocol (20)		X	Web of Science, iCite	61
**Total citation searching results**				987

© 2023 Marc I et al. *JAMA Network Open.*

eTable 8. Status of protocols for citation searching

Registration number	Title of the trial	Recruitment status
ACTRN12612000503820	In preterm infants born at <29 weeks gestation is an emulsion containing a higher amount of docosahexaenoic acid (DHA) more effective than an emulsion with no additional DHA in reducing the incidence of bronchopulmonary dysplasia at 36 weeks post menstrual age?	Completed
ACTRN12617001078347	Double-blind RCT of fish oil supplementation in pregnancy and lactation to improve metabolic health in the children of mothers with overweight or obesity	Active, not recruiting
N/A	Effect of alpha-linolenic acid intake and postmenstrual age on arachidonic and docosahexaenoic acid biosynthesis in preterm infants	N/A
NCT00226187	A Randomized Clinical Trial on Supplementation of DHA and AA to Preterm Infants	Completed
NCT00872664	Skin and Serum Carotenoids in Preterm Infants Fed on a Formula Supplemented With Carotenoids	Completed
NCT01103219	Nutrition, Growth and Development Among Very Preterm Infants	Terminated (a planned interim-analysis revealed increased number of infections in the intervention arm)
NCT01306838	Early Provision of Enteral Microlipid and Fish Oil to Infants With Enterostomy	Completed
NCT01732874	DHA Supplementation for Lactating Mothers	Completed
NCT01940640	Effect of Mother DHA Supplementation on Premature Newborn	Completed
NCT01955044	PUFA Supplementation in Premature Infants	Completed
NCT02371460	Maternal Omega-3 Supplementation to Reduce Bronchopulmonary Dysplasia	Active, not recruiting
NCT03192839	Early DHA Supplementation in Very Low Birth Weight Infants	Completed
NCT03201588	Multi-Center Study to Determine the Role of Fatty Acids in Serum in Preventing Retinopathy of Prematurity (MDM)	Completed
NCT03555019	Nutrition Therapy in the Immature Infant (ImNuT)	Active, not recruiting
NCT03700957	The Impact of Docosahexaenoic Acid on the Prevention of Necrotizing Enterocolitis in Preterm Neonates	Unknown
NCT03739463	Pro-omega-3, Reduction of Inflammation and Modulation of Prematurity	Unknown
NCT04746885	Effect of DHA on Proinflammatory Cytokines Including Platelets Activating Factor (PAF) in Preterm Neonates	Completed
ISRCTN59878178	Exploratory randomised double-blind controlled trial of breast milk fortifier with and without long chain polyunsaturated fatty acid (LCPUFA) supplementation on body composition in preterm infants	No longer recruiting

© 2023 Marc I et al. *JAMA Network Open.*

eTable 9. Reasons for exclusion at full-text assessment

Study	Reason for exclusion
**AbouElFadl et al,[1] 2021**	Does not exclusively include infants <29 weeks' gestation and does not report data stratified for <29 weeks' gestation
**Atwell et al,[2] 2013**	No data on BPD or death for the relevant population. Same dataset as Manley et al,[3] 2011 (included in this review)
**Baack et al,[4] 2016**	Does not exclusively include infants <29 weeks' gestation and does not report data stratified for <29 weeks' gestation
**Bernabe-García et al,[5] 2019**	Does not exclusively include infants <29 weeks' gestation and does not report data stratified for <29 weeks' gestation
**Bernabe-García et al,[6] 2021**	Does not exclusively include infants <29 weeks' gestation and does not report data stratified for <29 weeks' gestation
**Carlson et al,[7] 1996**	Intervention evaluates low doses of DHA
**Carlson et al,[8] 1998**	Intervention evaluates low doses of DHA
**Clandinin et al,[9] 1997**	Does not exclusively include infants <29 weeks' gestation and does not report data stratified for <29 weeks' gestation, no data on BPD or death
**Clandinin et al,[10] 2005**	Intervention evaluates low doses of DHA
**Fewtrell et al,[11] 2002**	Intervention evaluates low doses of DHA
**Frost et al,[12] 2021**	Does not exclusively include infants <29 weeks' gestation and does not report data stratified for <29 weeks' gestation
**Khalesi et al,[13] 2018**	Does not exclusively include infants <29 weeks' gestation and does not report data stratified for <29 weeks' gestation, no data on BPD or death
**Makrides et al,[14] 2009**	No data on BPD or death for the relevant population. Same dataset as Manley et al,[3] 2011 (included in this review)
**Manley et al,[15] 2010**	Conference abstract and same dataset as Manley et al,[3] 2011 (included in this review)
**Moltu et al,[16] 2014**	Does not exclusively include infants <29 weeks' gestation and does not report data stratified for <29 weeks' gestation
**Osman et al,[17] 2021**	Does not exclusively include infants <29 weeks' gestation and does not report data stratified for <29 weeks' gestation
**Picaud et al,[18] 2022**	Conference abstract
**Rigo et al,[19] 2017**	Does not exclusively include infants <29 weeks' gestation and does not report data stratified for <29 weeks' gestation
**Valentine et al,[20] 2019**	No clinical outcome
**Vanderhoof et al,[21] 2000**	Intervention evaluates low doses of DHA
**Strømmen et al,[22] 2016**	Not an RCT and same dataset as Moltu et al,[16] 2014 (excluded)
**Strømmen et al,[23] 2017**	Does not exclusively include infants <29 weeks' gestation and does not report data stratified for <29 weeks' gestation Same dataset as Moltu et al,[16] 2014 (excluded)
**Westerberg et al,[24] 2011**	Does not exclusively include infants <29 weeks' gestation and does not report data stratified for <29 weeks' gestation, no data on BPD or death

BPD: Bronchopulmonary dysplasia; DHA: Docosahexaenoic acid; RCT: Randomized Controlled Trial.

© 2023 Marc I et al. *JAMA Network Open.*

eFigure 1. Risk of bias assessment of included trials

	Randomization process	Deviations from the intended interventions	Missing outcome data	Measurement of the outcome	Selection of the reported result	Overall
Collins et al,[25] 2017	+	+	+	+	+	+
Hellström et al,[26] 2021	+	+	+	! [a]	+	!
Manley et al,[3] 2011	+	+	+	+	+	+
Marc et al,[27] 2020	+	+	+	+	+	+

+ Low; ! Some concerns

[a]Respiratory care management and consequently the assessment of BPD may have been influenced by the knowledge of the group due to the lack of placebo administration in the control group.

© 2023 Marc I et al. *JAMA Network Open.*

eFigure 2. Meta-analysis pooling risk ratio from reported frequencies on BPD outcomes

A BPD at 36 weeks' PMA

Study or Subgroup	Intervention Events	Intervention Total	Control Events	Control Total	Weight	Risk Ratio M-H, Random, 95% CI
**3.1.1 Physiological BPD based on oximetry[28]**						
Collins et al,[25] 2017	291	592	269	613	32.4%	1.12 [0.99, 1.26]
Marc et al,[27] 2020	105	252	72	229	24.5%	1.33 [1.04, 1.69]
**Subtotal (95% CI)**		**844**		**842**	**56.9%**	**1.18 [1.01, 1.37]**
Total events	396		341			
Heterogeneity: Tau² = 0.00; Chi² = 1.51, df = 1 (P = 0.22); I² = 34%						
Test for overall effect: Z = 2.12 (P = 0.03)						
**3.1.2 BPD with no oximetry assessment**						
Hellström et al,[26] 2021	48	101	48	105	21.2%	1.04 [0.78, 1.39]
Manley et al,[3] 2011	50	145	70	149	21.9%	0.73 [0.55, 0.97]
**Subtotal (95% CI)**		**246**		**254**	**43.1%**	**0.87 [0.62, 1.23]**
Total events	98		118			
Heterogeneity: Tau² = 0.04; Chi² = 2.84, df = 1 (P = 0.09); I² = 65%						
Test for overall effect: Z = 0.79 (P = 0.43)						
**Total (95% CI)**		**1090**		**1096**	**100.0%**	**1.05 [0.85, 1.29]**
Total events	494		459			
Heterogeneity: Tau² = 0.03; Chi² = 10.39, df = 3 (P = 0.02); I² = 71%						
Test for overall effect: Z = 0.44 (P = 0.66)						
Test for subgroup differences: Chi² = 2.50, df = 1 (P = 0.11), I² = 60.0%						

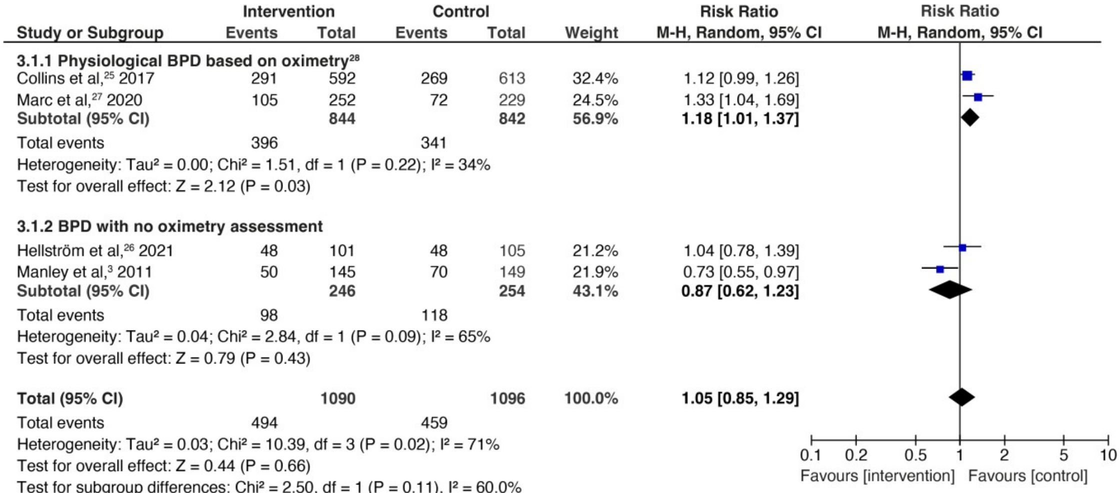

B "BPD or death" at 36 weeks' PMA[a]

Study or Subgroup	Intervention Events	Intervention Total	Control Events	Control Total	Weight	Risk Ratio M-H, Random, 95% CI
**3.2.1 Physiological BPD based on oximetry[28]**						
Collins et al,[25] 2017	330	631	298	642	34.4%	1.13 [1.01, 1.26]
Marc et al,[27] 2020	121	268	98	255	24.2%	1.17 [0.96, 1.44]
**Subtotal (95% CI)**		**899**		**897**	**58.7%**	**1.14 [1.03, 1.25]**
Total events	451		396			
Heterogeneity: Tau² = 0.00; Chi² = 0.12, df = 1 (P = 0.72); I² = 0%						
Test for overall effect: Z = 2.58 (P = 0.010)						
**3.2.2 BPD with no oximetry assessment**						
Hellström et al,[26] 2021	64	101	61	106	22.6%	1.10 [0.88, 1.37]
Manley et al,[3] 2011	55	147	73	149	18.7%	0.76 [0.59, 1.00]
**Subtotal (95% CI)**		**248**		**255**	**41.3%**	**0.92 [0.64, 1.33]**
Total events	119		134			
Heterogeneity: Tau² = 0.05; Chi² = 4.47, df = 1 (P = 0.03); I² = 78%						
Test for overall effect: Z = 0.43 (P = 0.67)						
**Total (95% CI)**		**1147**		**1152**	**100.0%**	**1.05 [0.90, 1.23]**
Total events	570		530			
Heterogeneity: Tau² = 0.01; Chi² = 7.76, df = 3 (P= 0.05); I² = 61%						
Test for overall effect: Z = 0.65 (P = 0.51)						
Test for subgroup differences: Chi² = 1.16, df = 1 (P = 0.28), I² = 14.2%						

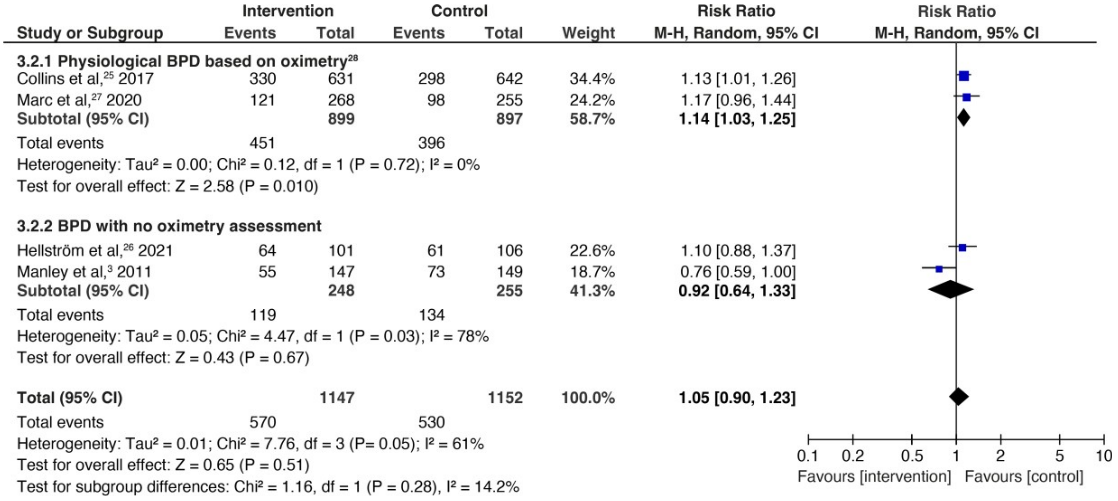

C Death up to 36 weeks' PMA[a]

Study or Subgroup	Intervention Events	Intervention Total	Control Events	Control Total	Weight	Risk Ratio M-H, Random, 95% CI
Collins et al,[25] 2017	39	631	29	642	35.8%	1.37 [0.86, 2.18]
Hellström et al,[26] 2021	16	101	13	106	25.7%	1.29 [0.65, 2.55]
Manley et al,[3] 2011	5	145	3	149	9.3%	1.71 [0.42, 7.04]
Marc et al,[27] 2020	16	268	26	255	29.2%	0.59 [0.32, 1.07]
**Total (95% CI)**		**1145**		**1152**	**100.0%**	**1.07 [0.67, 1.72]**
Total events	76		71			
Heterogeneity: Tau² = 0.10; Chi² = 5.68, df = 3 (P= 0.13); I² = 47%						
Test for overall effect: Z = 0.30 (P = 0.77)						

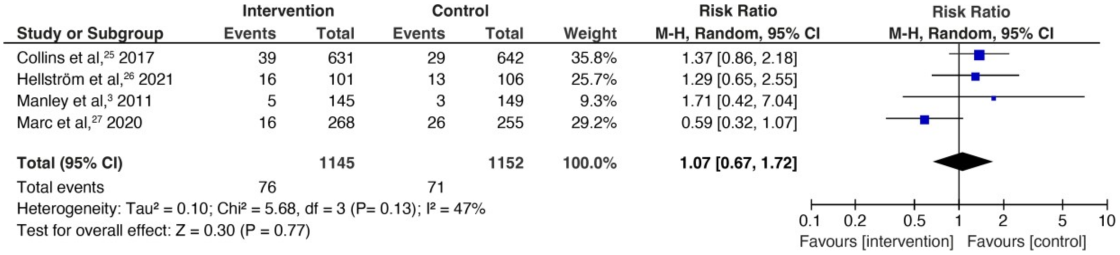

© 2023 Marc I et al. *JAMA Network Open.*

D Moderate-to-severe BPD at 36 weeks' PMA[b]

Study or Subgroup	Intervention Events	Intervention Total	Control Events	Control Total	Weight	Risk Ratio M-H, Random, 95% CI
Collins et al,[25] 2017	267	592	244	612	66.8%	1.13 [0.99, 1.29]
Hellström et al,[26] 2021	48	101	48	106	13.5%	1.05 [0.78, 1.41]
Marc et al,[27] 2020	104	252	71	229	19.7%	1.33 [1.04, 1.70]
**Total (95% CI)**		**945**		**947**	**100.0%**	**1.16 [1.04, 1.29]**
Total events	419		363			

Heterogeneity: Tau² = 0.00; Chi² = 1.82, df = 2 (P= 0.40); I² = 0%
Test for overall effect: Z = 2.64 (P = 0.008)

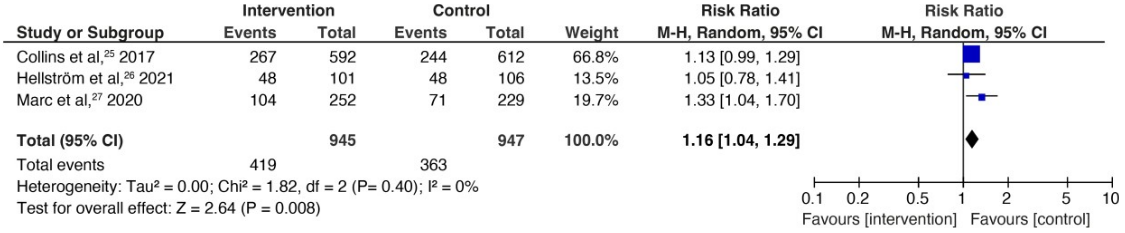

E Severe BPD at 36 weeks' PMA[b]

Study or Subgroup	Intervention Events	Intervention Total	Control Events	Control Total	Weight	Risk Ratio M-H, Random, 95% CI
Collins et al,[25] 2017	202	592	194	612	64.5%	1.08 [0.92, 1.26]
Hellström et al,[26] 2021	22	101	21	106	8.3%	1.10 [0.65, 1.87]
Marc et al,[27] 2020	88	252	58	229	27.2%	1.38 [1.04, 1.82]
**Total (95% CI)**		**945**		**947**	**100.0%**	**1.15 [0.99, 1.35]**
Total events	312		273			

Heterogeneity: Tau² = 0.00; Chi² = 2.29, df = 2 (P= 0.32); I² = 13%
Test for overall effect: Z = 1.79 (P = 0.07)

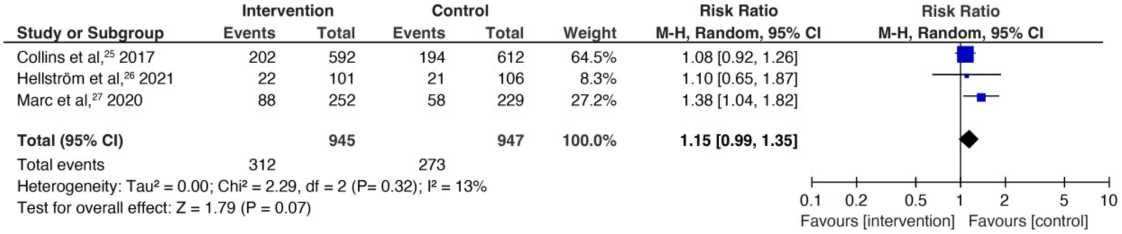

BPD: Bronchopulmonary dysplasia; CI: Confidence interval; M-H: Mantel-Haenszel; PMA: Postmenstrual age.
[a]Death up to 36 weeks' PMA except in Hellström et al,[26] 2021 where death was up to 40 weeks' PMA.
[b]Classified according to criteria from the National Institute of Child Health and Human Development[29] in Collins et al,[25] 2017 and Marc et al,[27] 2020. Definition not specified in Hellström et al,[26] 2021.

© 2023 Marc I et al. *JAMA Network Open.*

eFigure 3. Meta-analysis results on BPD outcome stratified by sex

A BPD at 36 weeks' PMA[a]

Study or Subgroup	log[Risk Ratio]	SE	Intervention Total	Control Total	Weight	Risk Ratio IV, Random, 95% CI
**4.1.1 Females**						
Collins et al,[25] 2017	0.157	0.0852	277	287	21.8%	1.17 [0.99, 1.38]
Manley et al,[3] 2011	-0.1278	0.2216	67	73	11.4%	0.88 [0.57, 1.36]
Marc et al,[27] 2020	0.5227	0.1786	123	109	14.2%	1.69 [1.19, 2.39]
**Subtotal (95% CI)**			**467**	**469**	**47.4%**	**1.22 [0.90, 1.64]**
Heterogeneity: Tau² = 0.04; Chi² = 5.67, df = 2 (P= 0.06); I² = 65%						
Test for overall effect: Z = 1.29 (P = 0.20)						
**4.1.2 Males**						
Collins et al,[25] 2017	0.0953	0.0642	315	326	23.4%	1.10 [0.97, 1.25]
Manley et al,[3] 2011	-0.462	0.1717	75	73	14.7%	0.63 [0.45, 0.88]
Marc et al,[27] 2020	0.0427	0.1733	129	120	14.5%	1.04 [0.74, 1.47]
**Subtotal (95% CI)**			**519**	**519**	**52.6%**	**0.92 [0.66, 1.27]**
Heterogeneity: Tau² = 0.06; Chi² = 9.25, df = 2 (P = 0.010); I² = 78%						
Test for overall effect: Z = 0.51 (P = 0.61)						
**Total (95% CI)**			**986**	**988**	**100.0%**	**1.06 [0.87, 1.28]**

Heterogeneity: Tau² = 0.04; Chi² = 17.87, df = 5 (P = 0.003); I² = 72%
Test for overall effect: Z = 0.55 (P = 0.58)
Test for subgroup differences: Chi² = 1.57, df = 1 (P = 0.21), I² = 36.2%

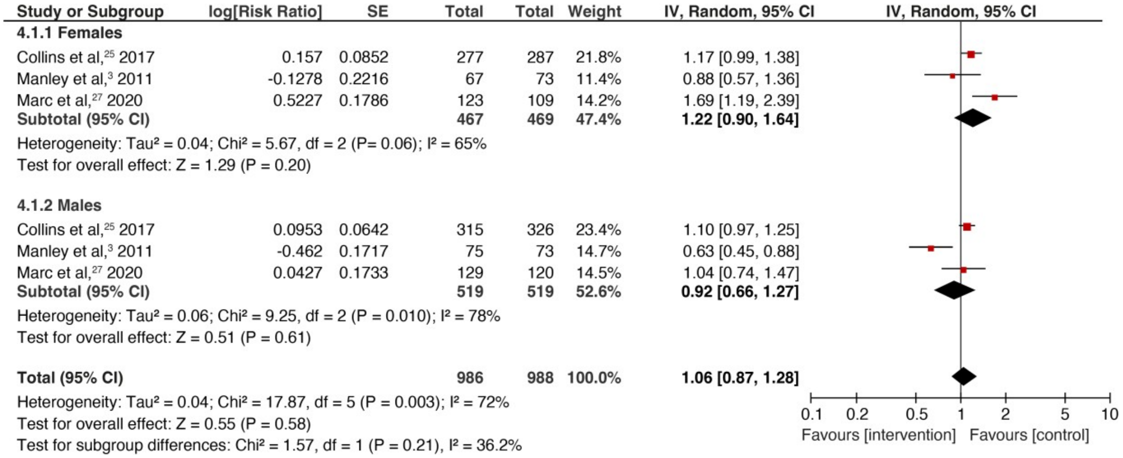

B "BPD or death" at 36 weeks' PMA[a]

Study or Subgroup	log[Risk Ratio]	SE	Intervention Total	Control Total	Weight	Risk Ratio IV, Random, 95% CI
**4.2.1 Females**						
Collins et al,[25] 2017	0.1398	0.0868	293	300	21.5%	1.15 [0.97, 1.36]
Manley et al,[3] 2011	-0.0943	0.2125	70	75	10.9%	0.91 [0.60, 1.38]
Marc et al,[27] 2020	0.4256	0.1533	133	118	15.2%	1.53 [1.13, 2.07]
**Subtotal (95% CI)**			**496**	**493**	**47.6%**	**1.20 [0.94, 1.52]**
Heterogeneity: Tau² = 0.03; Chi² = 4.44, df = 2 (P= 0.11); I² = 55%						
Test for overall effect: Z = 1.44 (P = 0.15)						
**4.2.2 Males**						
Collins et al,[25] 2017	0.0862	0.0701	338	342	23.1%	1.09 [0.95, 1.25]
Manley et al,[3] 2011	-0.4155	0.1732	77	74	13.6%	0.66 [0.47, 0.93]
Marc et al,[27] 2020	-0.0945	0.1475	135	137	15.7%	0.91 [0.68, 1.21]
**Subtotal (95% CI)**			**550**	**553**	**52.4%**	**0.90 [0.68, 1.19]**
Heterogeneity: Tau² = 0.05; Chi² = 7.68, df = 2 (P = 0.02); I² = 74%						
Test for overall effect: Z = 0.75 (P = 0.45)						
**Total (95% CI)**			**1046**	**1046**	**100.0%**	**1.03 [0.86, 1.24]**

Heterogeneity: Tau² = 0.03; Chi² = 15.78, df = 5 (P = 0.008); I² = 68%
Test for overall effect: Z = 0.36 (P = 0.72)
Test for subgroup differences: Chi² = 2.28, df = 1 (P = 0.13), I² = 56.1%

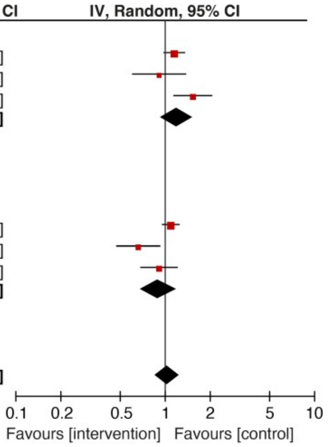

C Death up to 36 weeks' PMA

Study or Subgroup	log[Risk Ratio]	SE	Intervention Total	Control Total	Weight	Risk Ratio IV, Random, 95% CI
**4.3.1 Females**						
Collins et al,[25] 2017	0.1823	0.3798	293	300	23.1%	1.20 [0.57, 2.53]
Manley et al,[3] 2011	0.4745	0.8976	70	75	6.2%	1.61 [0.28, 9.33]
Marc et al,[27] 2020	-0.0143	0.4417	133	118	19.1%	0.99 [0.41, 2.34]
**Subtotal (95% CI)**			**496**	**493**	**48.5%**	**1.14 [0.67, 1.96]**
Heterogeneity: Tau² = 0.00; Chi² = 0.27, df = 2 (P = 0.87); I² = 0%						
Test for overall effect: Z = 0.49 (P = 0.63)						
**4.3.2 Males**						
Collins et al,[25] 2017	0.3646	0.2999	338	342	29.8%	1.44 [0.80, 2.59]
Manley et al,[3] 2011	0.6534	1.2139	77	74	3.6%	1.92 [0.18, 20.75]
Marc et al,[27] 2020	-1.0267	0.4591	135	137	18.2%	0.36 [0.15, 0.88]
**Subtotal (95% CI)**			**550**	**553**	**51.5%**	**0.88 [0.29, 2.67]**
Heterogeneity: Tau² = 0.62; Chi² = 6.76, df = 2 (P = 0.03); I² = 70%						
Test for overall effect: Z = 0.23 (P = 0.82)						
**Total (95% CI)**			**1046**	**1046**	**100.0%**	**1.01 [0.64, 1.61]**

Heterogeneity: Tau² = 0.10; Chi² = 7.21, df = 5 (P = 0.21); I² = 31%
Test for overall effect: Z = 0.06 (P = 0.95)
Test for subgroup differences: Chi² = 0.18, df = 1 (P = 0.67), I² = 0%

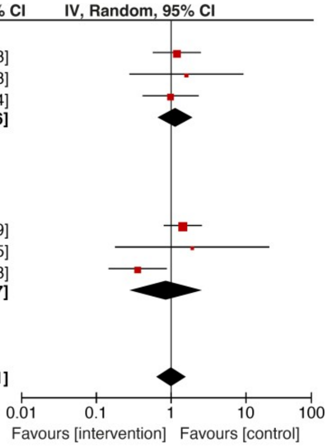

© 2023 Marc I et al. *JAMA Network Open.*

D Moderate-to-severe BPD at 36 weeks' PMA[b]

Study or Subgroup	log[Risk Ratio]	SE	Intervention Total	Control Total	Weight	Risk Ratio IV, Random, 95% CI
**4.4.1 Females**						
Collins et al,[25] 2017	0.1653	0.1072	277	287	30.6%	1.18 [0.96, 1.46]
Marc et al,[27] 2020	0.5056	0.1794	123	109	15.0%	1.66 [1.17, 2.36]
**Subtotal (95% CI)**			**400**	**396**	**45.6%**	**1.36 [0.98, 1.88]**
Heterogeneity: Tau² = 0.04; Chi² = 2.65, df = 1 (P = 0.10); I² = 62%						
Test for overall effect: Z = 1.82 (P = 0.07)						
**4.4.2 Males**						
Collins et al,[25] 2017	0.0954	0.0856	315	326	38.8%	1.10 [0.93, 1.30]
Marc et al,[27] 2020	0.0674	0.1751	129	120	15.6%	1.07 [0.76, 1.51]
**Subtotal (95% CI)**			**444**	**446**	**54.4%**	**1.09 [0.94, 1.27]**
Heterogeneity: Tau² = 0.00; Chi² = 0.02, df = 1 (P = 0.89); I² = 0%						
Test for overall effect: Z = 1.17 (P = 0.24)						
**Total (95% CI)**			**844**	**842**	**100.0%**	**1.19 [1.02, 1.39]**
Heterogeneity: Tau² = 0.01; Chi² = 4.56, df = 3 (P = 0.21); I² = 34%						
Test for overall effect: Z = 2.23 (P = 0.03)						
Test for subgroup differences: Chi² = 1.36, df = 1 (P = 0.24), I² = 26.6%						

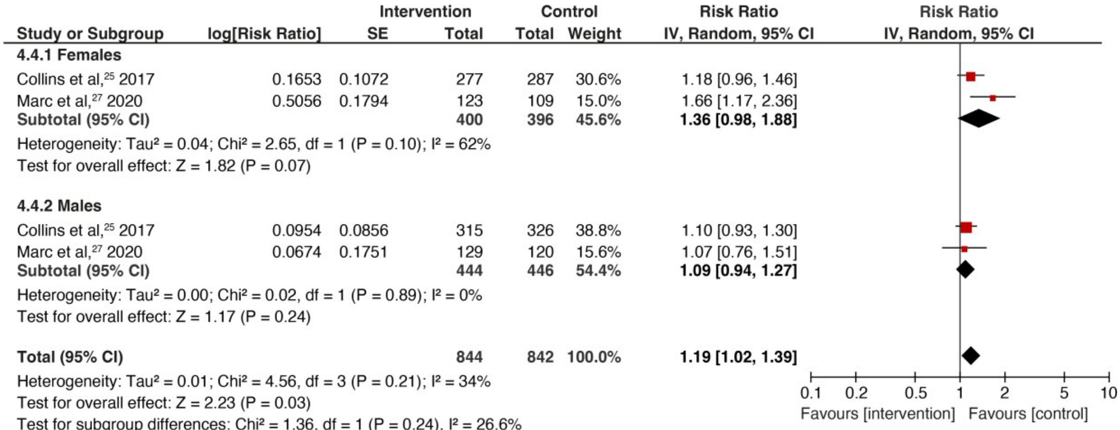

E Severe BPD at 36 weeks' PMA[b]

Study or Subgroup	log[Risk Ratio]	SE	Intervention Total	Control Total	Weight	Risk Ratio IV, Random, 95% CI
**4.5.1 Females**						
Collins et al,[25] 2017	0.0953	0.1139	277	286	31.7%	1.10 [0.88, 1.38]
Marc et al,[27] 2020	0.4953	0.1993	123	109	13.8%	1.64 [1.11, 2.43]
**Subtotal (95% CI)**			**400**	**395**	**45.5%**	**1.30 [0.88, 1.91]**
Heterogeneity: Tau² = 0.05; Chi² = 3.04, df = 1 (P = 0.08); I² = 67%						
Test for overall effect: Z = 1.33 (P = 0.18)						
**4.5.2 Males**						
Collins et al,[25] 2017	0.0488	0.0901	315	326	41.5%	1.05 [0.88, 1.25]
Marc et al,[27] 2020	0.1313	0.2061	129	120	13.0%	1.14 [0.76, 1.71]
**Subtotal (95% CI)**			**444**	**446**	**54.5%**	**1.06 [0.91, 1.25]**
Heterogeneity: Tau² = 0.00; Chi² = 0.13, df = 1 (P = 0.71); I² = 0%						
Test for overall effect: Z = 0.75 (P = 0.45)						
**Total (95% CI)**			**844**	**841**	**100.0%**	**1.15 [0.98, 1.34]**
Heterogeneity: Tau² = 0.01; Chi² = 4.22, df = 3 (P = 0.24); I² = 29%						
Test for overall effect: Z = 1.68 (P = 0.09)						
Test for subgroup differences: Chi² = 0.87, df = 1 (P = 0.35), I² = 0%						

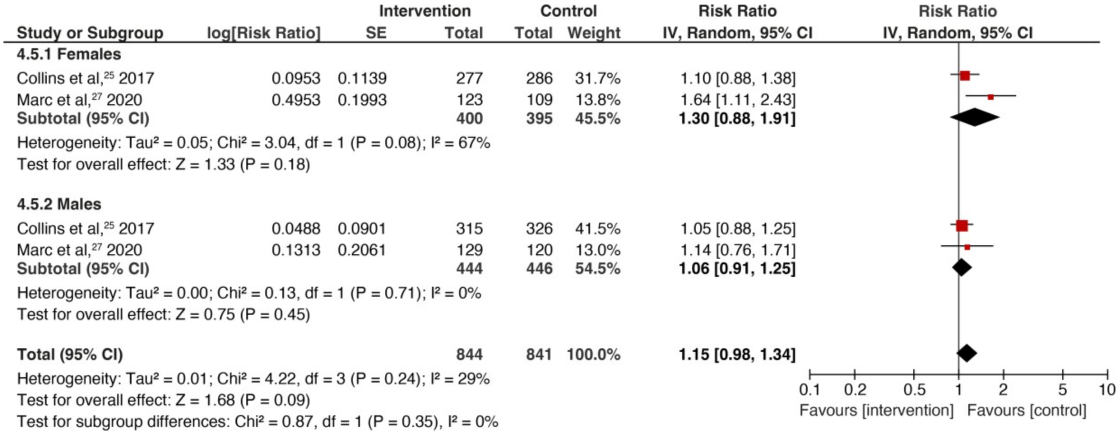

BPD: Bronchopulmonary dysplasia; CI: Confidence interval; IV: Inverse variance; PMA: Postmenstrual age; SE: Standard error.

[a]Physiological BPD based on oximetry[28] except in Manley et al,[3] 2011 where BPD was with no oximetry assessment.

[b]Classified according to criteria from the National Institute of Child Health and Human Development.[29]

© 2023 Marc I et al. *JAMA Network Open.*

eFigure 4. Meta-analysis results on BPD outcome stratified by gestational age

A BPD at 36 weeks' PMA[a]

Study or Subgroup	log[Risk Ratio]	SE	Intervention Total	Control Total	Weight	Risk Ratio IV, Random, 95% CI
**5.1.1 Born before 27 weeks' gestation**						
Collins et al,[25] 2017	0.131	0.0567	289	292	21.2%	1.14 [1.02, 1.27]
Hellström et al,[26] 2021	-0.049	0.1568	78	86	13.6%	0.95 [0.70, 1.29]
Manley et al,[3] 2011	-0.2357	0.1489	50	51	14.1%	0.79 [0.59, 1.06]
Marc et al,[27] 2020	0.0967	0.127	125	121	15.8%	1.10 [0.86, 1.41]
**Subtotal (95% CI)**			**542**	**550**	**64.6%**	**1.02 [0.87, 1.20]**
Heterogeneity: Tau² = 0.01; Chi² = 5.98, df = 3 (P = 0.11); I² = 50%						
Test for overall effect: Z = 0.28 (P = 0.78)						
**5.1.2 Born at or after 27 weeks' gestation**						
Collins et al,[25] 2017	0.077	0.1222	303	321	16.2%	1.08 [0.85, 1.37]
Hellström et al,[26] 2021	0.7252	0.5039	23	19	2.8%	2.07 [0.77, 5.54]
Manley et al,[3] 2011	-0.4005	0.2263	92	95	9.4%	0.67 [0.43, 1.04]
Marc et al,[27] 2020	0.8624	0.2827	127	108	7.0%	2.37 [1.36, 4.12]
**Subtotal (95% CI)**			**545**	**543**	**35.4%**	**1.27 [0.76, 2.12]**
Heterogeneity: Tau² = 0.20; Chi² = 13.73, df = 3 (P = 0.003); I² = 78%						
Test for overall effect: Z = 0.91 (P = 0.36)						
**Total (95% CI)**			**1087**	**1093**	**100.0%**	**1.06 [0.89, 1.26]**
Heterogeneity: Tau² = 0.03; Chi² = 19.81, df = 7 (P = 0.006); I² = 65%						
Test for overall effect: Z = 0.66 (P = 0.51)						
Test for subgroup differences: Chi² = 0.62, df = 1 (P = 0.43), I² = 0%						

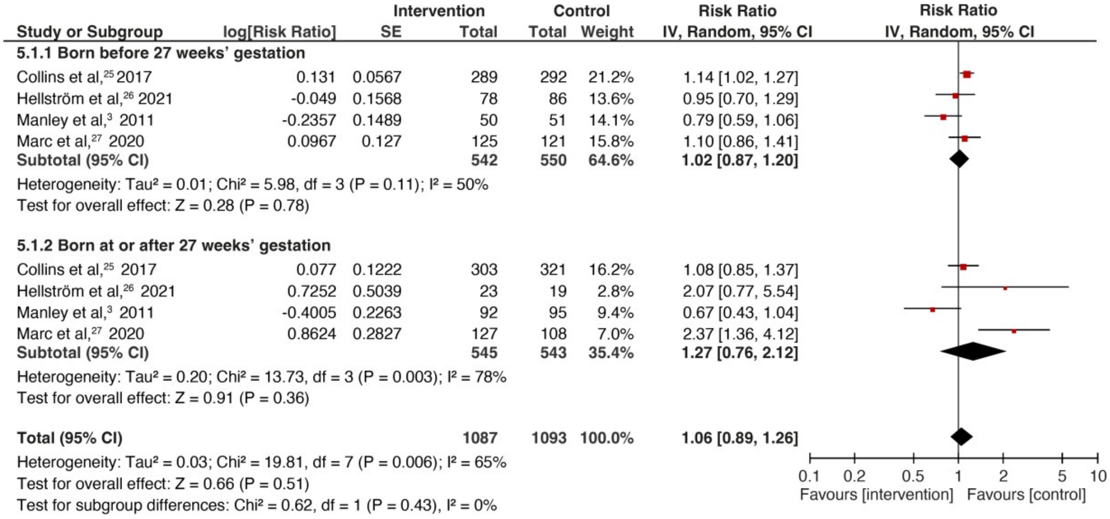

B "BPD or death" at 36 weeks' PMA[a, b]

Study or Subgroup	log[Risk Ratio]	SE	Intervention Total	Control Total	Weight	Risk Ratio IV, Random, 95% CI
**5.2.1 Born before 27 weeks' gestation**						
Collins et al,[25] 2017	0.1133	0.0527	322	317	21.4%	1.12 [1.01, 1.24]
Hellström et al,[26] 2021	0.0198	0.1114	78	87	16.2%	1.02 [0.82, 1.27]
Manley et al,[3] 2011	-0.2231	0.1468	52	53	13.2%	0.80 [0.60, 1.07]
Marc et al,[27] 2020	0.0072	0.102	139	147	17.1%	1.01 [0.82, 1.23]
**Subtotal (95% CI)**			**591**	**604**	**67.8%**	**1.02 [0.90, 1.16]**
Heterogeneity: Tau² = 0.01; Chi² = 5.13, df = 3 (P = 0.16); I² = 42%						
Test for overall effect: Z = 0.36 (P = 0.72)						
**5.2.2 Born at or after 27 weeks' gestation**						
Collins et al,[25] 2017	0.0953	0.1197	309	325	15.5%	1.10 [0.87, 1.39]
Hellström et al,[26] 2021	0.9083	0.4896	23	19	2.4%	2.48 [0.95, 6.47]
Manley et al,[3] 2011	-0.3147	0.2247	95	96	8.3%	0.73 [0.47, 1.13]
Marc et al,[27] 2020	0.8968	0.2807	129	108	6.1%	2.45 [1.41, 4.25]
**Subtotal (95% CI)**			**556**	**548**	**32.2%**	**1.36 [0.81, 2.27]**
Heterogeneity: Tau² = 0.20; Chi² = 13.96, df = 3 (P = 0.003); I² = 79%						
Test for overall effect: Z = 1.18 (P = 0.24)						
**Total (95% CI)**			**1147**	**1152**	**100.0%**	**1.07 [0.91, 1.25]**
Heterogeneity: Tau² = 0.03; Chi² = 19.83, df = 7 (P = 0.006); I² = 65%						
Test for overall effect: Z = 0.80 (P = 0.42)						
Test for subgroup differences: Chi² = 1.12, df = 1 (P = 0.29), I² = 10.9%						

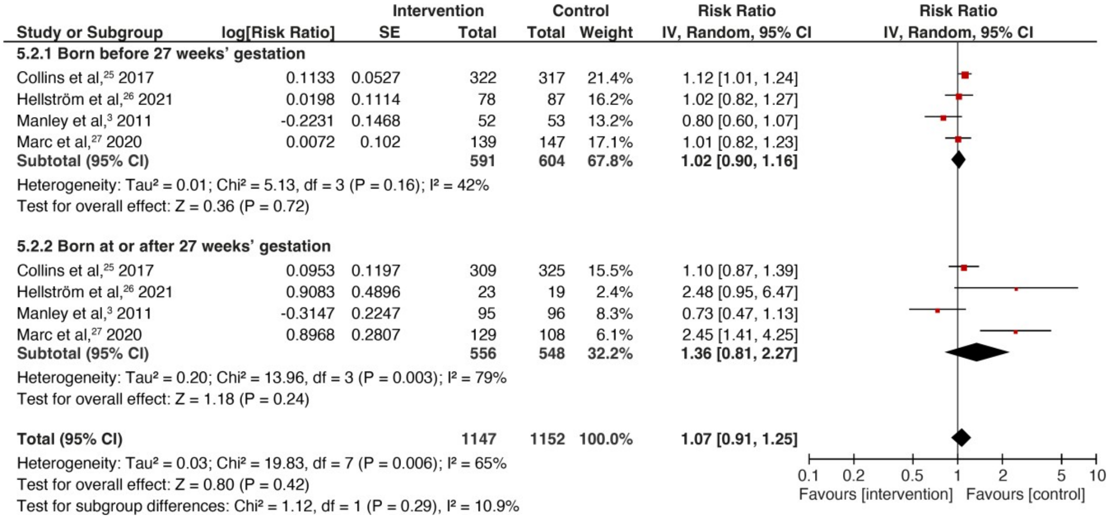

© 2023 Marc I et al. *JAMA Network Open.*

C Death up to 36 weeks' PMA[b]

Study or Subgroup	log[Risk Ratio]	SE	Intervention Total	Control Total	Weight	Risk Ratio IV, Random, 95% CI
**5.3.1 Born before 27 weeks' gestation**						
Collins et al,[25] 2017	0.2624	0.2541	322	317	36.3%	1.30 [0.79, 2.14]
Hellström et al,[26] 2021	0.1833	0.3522	78	87	21.5%	1.20 [0.60, 2.40]
Manley et al,[3] 2011	0.019	0.9808	52	53	3.2%	1.02 [0.15, 6.97]
Marc et al,[27] 2020	-0.5631	0.3097	139	147	26.7%	0.57 [0.31, 1.04]
**Subtotal (95% CI)**			**591**	**604**	**87.7%**	**0.98 [0.63, 1.52]**
Heterogeneity: Tau² = 0.07; Chi² = 4.64, df = 3 (P = 0.20); I² = 35%						
Test for overall effect: Z = 0.11 (P = 0.91)						
**5.3.2 Born at or after 27 weeks' gestation**						
Collins et al,[25] 2017	0.4511	0.6376	309	325	7.3%	1.57 [0.45, 5.48]
Hellström et al,[26] 2021	1.4271	1.5193	23	19	1.3%	4.17 [0.21, 81.85]
Manley et al,[3] 2011	1.1091	1.1456	95	96	2.4%	3.03 [0.32, 28.63]
Marc et al,[27] 2020	1.4333	1.5437	129	108	1.3%	4.19 [0.20, 86.39]
**Subtotal (95% CI)**			**556**	**548**	**12.3%**	**2.18 [0.83, 5.75]**
Heterogeneity: Tau² = 0.00; Chi² = 0.71, df = 3 (P = 0.87); I² = 0%						
Test for overall effect: Z = 1.57 (P = 0.12)						
**Total (95% CI)**			**1147**	**1152**	**100.0%**	**1.09 [0.77, 1.54]**
Heterogeneity: Tau² = 0.02; Chi² = 7.62, df = 7 (P= 0.37); I² = 8%						
Test for overall effect: Z = 0.46 (P = 0.64)						
Test for subgroup differences: Chi² = 2.18, df = 1 (P = 0.14), I² = 54.1%						

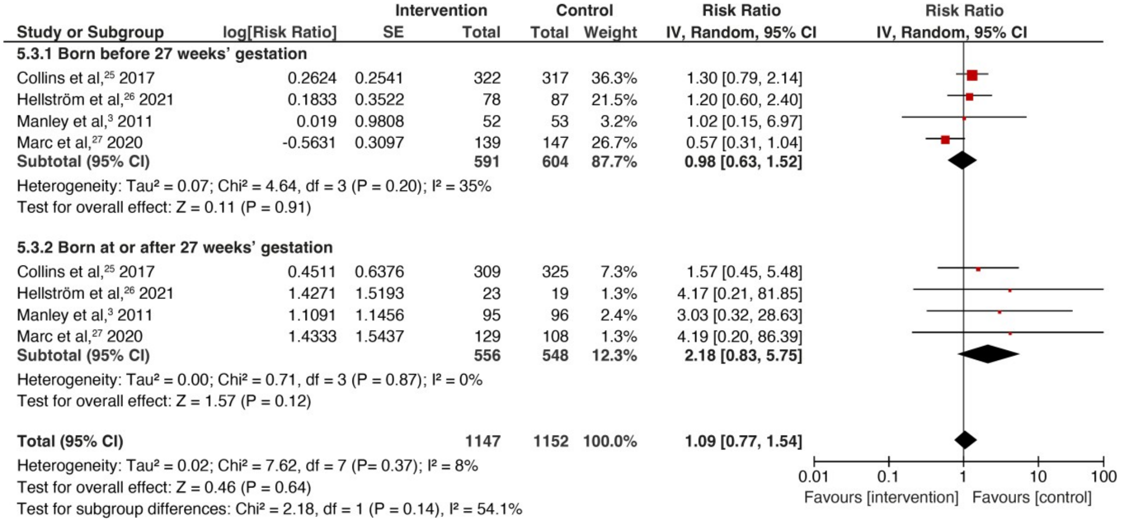

D Moderate-to-severe BPD at 36 weeks' PMA[c]

Study or Subgroup	log[Risk Ratio]	SE	Intervention Total	Control Total	Weight	Risk Ratio IV, Random, 95% CI
**5.4.1 Born before 27 weeks' gestation**						
Collins et al,[25] 2017	0.1035	0.064	289	292	39.0%	1.11 [0.98, 1.26]
Marc et al,[27] 2020	0.0967	0.127	125	121	27.1%	1.10 [0.86, 1.41]
**Subtotal (95% CI)**			**414**	**413**	**66.1%**	**1.11 [0.99, 1.24]**
Heterogeneity: Tau² = 0.00; Chi² = 0.00, df = 1 (P = 0.96); I² = 0%						
Test for overall effect: Z = 1.79 (P = 0.07)						
**5.4.2 Born at or after 27 weeks' gestation**						
Collins et al,[25] 2017	0.1543	0.1457	303	321	24.1%	1.17 [0.88, 1.55]
Marc et al,[27] 2020	0.9106	0.2934	127	108	9.8%	2.49 [1.40, 4.42]
**Subtotal (95% CI)**			**430**	**429**	**33.9%**	**1.63 [0.78, 3.41]**
Heterogeneity: Tau² = 0.23; Chi² = 5.33, df = 1 (P = 0.02); I² = 81%						
Test for overall effect: Z = 1.30 (P = 0.19)						
**Total (95% CI)**			**844**	**842**	**100.0%**	**1.21 [0.99, 1.49]**
Heterogeneity: Tau² = 0.02; Chi² = 7.34, df = 3 (P = 0.06); I² = 59%						
Test for overall effect: Z = 1.86 (P = 0.06)						
Test for subgroup differences: Chi² = 1.04, df = 1 (P = 0.31), I² = 3.8%						

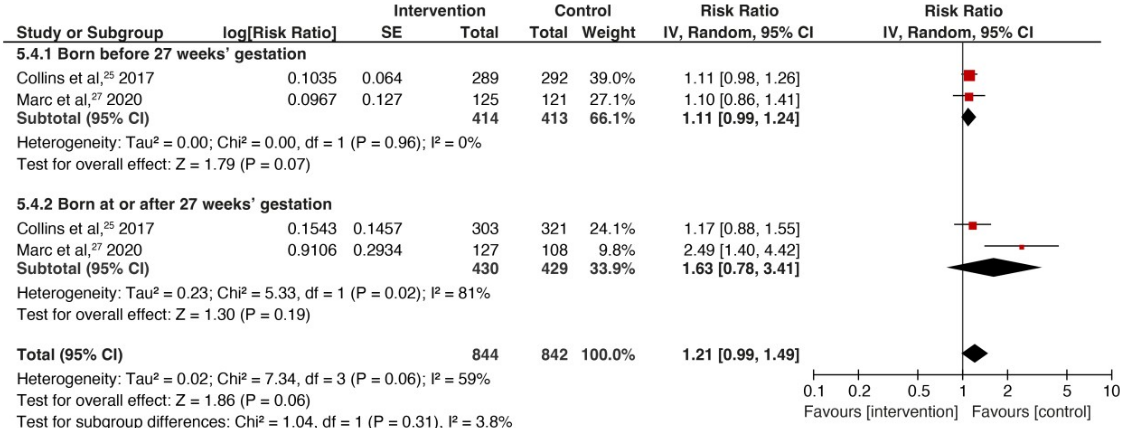

E Severe BPD at 36 weeks' PMA[c]

Study or Subgroup	log[Risk Ratio]	SE	Intervention Total	Control Total	Weight	Risk Ratio IV, Random, 95% CI
**5.5.1 Born before 27 weeks' gestation**						
Collins et al,[25] 2017	0.0488	0.0786	289	291	39.0%	1.05 [0.90, 1.22]
Marc et al,[27] 2020	0.1393	0.1487	125	121	27.4%	1.15 [0.86, 1.54]
**Subtotal (95% CI)**			**414**	**412**	**66.4%**	**1.07 [0.93, 1.23]**
Heterogeneity: Tau² = 0.00; Chi² = 0.29, df = 1 (P = 0.59); I² = 0%						
Test for overall effect: Z = 0.99 (P = 0.32)						
**5.5.2 Born at or after 27 weeks' gestation**						
Collins et al,[25] 2017	0.131	0.1744	303	321	23.7%	1.14 [0.81, 1.60]
Marc et al,[27] 2020	0.9693	0.3393	127	108	10.0%	2.64 [1.36, 5.13]
**Subtotal (95% CI)**			**430**	**429**	**33.6%**	**1.65 [0.73, 3.73]**
Heterogeneity: Tau² = 0.28; Chi² = 4.83, df = 1 (P = 0.03); I² = 79%						
Test for overall effect: Z = 1.20 (P = 0.23)						
**Total (95% CI)**			**844**	**841**	**100.0%**	**1.20 [0.95, 1.52]**
Heterogeneity: Tau² = 0.03; Chi² = 7.07, df = 3 (P = 0.07); I² = 58%						
Test for overall effect: Z = 1.53 (P = 0.13)						
Test for subgroup differences: Chi² = 1.04, df = 1 (P = 0.31), I² = 4.2%						

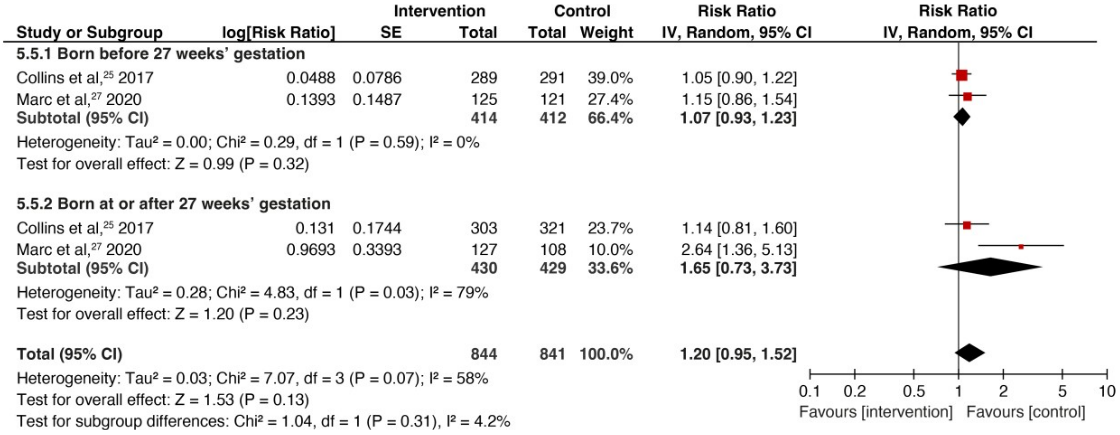

BPD: Bronchopulmonary dysplasia; CI: Confidence interval; IV: Inverse variance; PMA: Postmenstrual age; SE: Standard error.

[a]Physiological BPD based on oximetry[28] except in Hellström et al,[26] 2021 and Manley et al,[3] 2011 where BPD was with no oximetry assessment.

[b]Death up to 36 weeks' PMA except in Hellström et al,[26] 2021 where death was up to 40 weeks' PMA.

[c]Classified according to criteria from the National Institute of Child Health and Human Development.[29]

© 2023 Marc I et al. *JAMA Network Open.*

eFigure 5. Meta-analysis results on BPD outcome stratified by mode of administration, source of DHA and type of supplementation

A BPD at 36 weeks' PMA by mode of administration[a]

Study or Subgroup	log[Risk Ratio]	SE	Intervention Total	Control Total	Weight	Risk Ratio IV, Random, 95% CI
**6.1.1 Direct enteral supplementation**						
Collins et al,[25] 2017	0.1222	0.0523	592	613	34.4%	1.13 [1.02, 1.25]
Hellström et al,[26] 2021	0.1044	0.2069	101	105	16.4%	1.11 [0.74, 1.67]
**Subtotal (95% CI)**			**693**	**718**	**50.7%**	**1.13 [1.02, 1.25]**
Heterogeneity: Tau² = 0.00; Chi² = 0.01, df = 1 (P = 0.93); I² = 0%						
Test for overall effect: Z = 2.39 (P = 0.02)						
**6.1.2 Through breast milk/formula**						
Manley et al,[3] 2011	-0.2877	0.14	145	149	23.5%	0.75 [0.57, 0.99]
Marc et al,[27] 2020	0.3075	0.1224	252	229	25.7%	1.36 [1.07, 1.73]
**Subtotal (95% CI)**			**397**	**378**	**49.3%**	**1.01 [0.57, 1.82]**
Heterogeneity: Tau² = 0.16; Chi² = 10.24, df = 1 (P = 0.001); I² = 90%						
Test for overall effect: Z = 0.05 (P = 0.96)						
**Total (95% CI)**			**1090**	**1096**	**100.0%**	**1.07 [0.86, 1.34]**
Heterogeneity: Tau² = 0.03; Chi² = 10.71, df = 3 (P = 0.01); I² = 72%						
Test for overall effect: Z = 0.63 (P = 0.53)						
Test for subgroup differences: Chi² = 0.13, df = 1 (P = 0.72), I² = 0%						

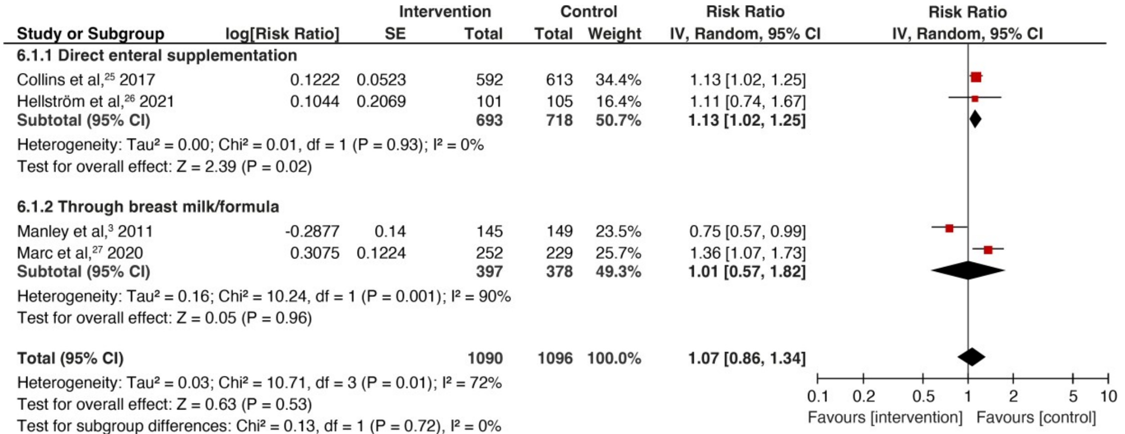

B BPD at 36 weeks' PMA by source of DHA[a]

Study or Subgroup	log[Risk Ratio]	SE	Intervention Total	Control Total	Weight	Risk Ratio IV, Random, 95% CI
**6.2.1 Algae oil**						
Hellström et al,[26] 2021	0.1044	0.2069	101	105	16.4%	1.11 [0.74, 1.67]
Marc et al,[27] 2020	0.3075	0.1224	252	229	25.7%	1.36 [1.07, 1.73]
**Subtotal (95% CI)**			**353**	**334**	**42.1%**	**1.29 [1.05, 1.59]**
Heterogeneity: Tau² = 0.00; Chi² = 0.71, df = 1 (P = 0.40); I² = 0%						
Test for overall effect: Z = 2.42 (P = 0.02)						
**6.2.2 Fish oil**						
Collins et al,[25] 2017	0.1222	0.0523	592	613	34.4%	1.13 [1.02, 1.25]
Manley et al,[3] 2011	-0.2877	0.14	145	149	23.5%	0.75 [0.57, 0.99]
**Subtotal (95% CI)**			**737**	**762**	**57.9%**	**0.94 [0.63, 1.40]**
Heterogeneity: Tau² = 0.07; Chi² = 7.52, df = 1 (P = 0.006); I² = 87%						
Test for overall effect: Z = 0.30 (P = 0.76)						
**Total (95% CI)**			**1090**	**1096**	**100.0%**	**1.07 [0.86, 1.34]**
Heterogeneity: Tau² = 0.03; Chi² = 10.71, df = 3 (P = 0.01); I² = 72%						
Test for overall effect: Z = 0.63 (P = 0.53)						
Test for subgroup differences: Chi² = 1.91, df = 1 (P = 0.17), I² = 47.6%						

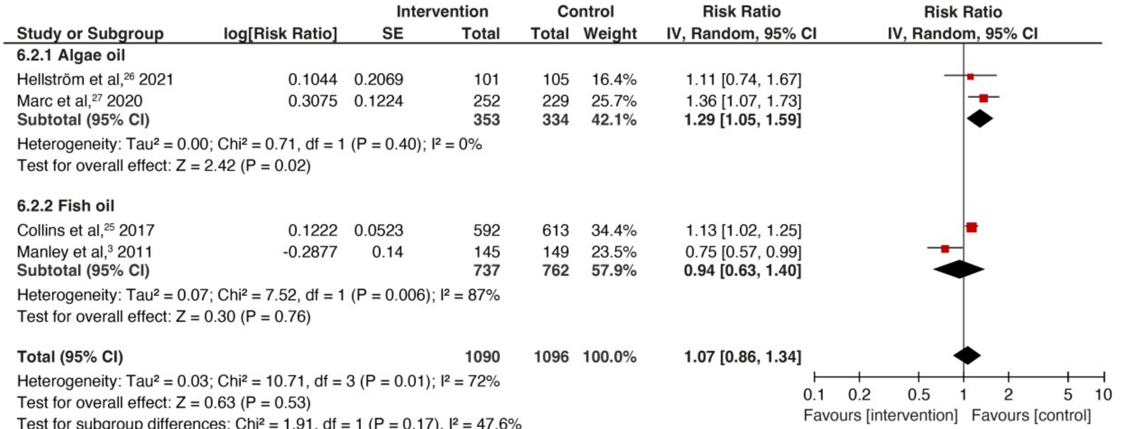

C BPD at 36 weeks' PMA by type of supplementation[a]

Study or Subgroup	log[Risk Ratio]	SE	Intervention Total	Control Total	Weight	Risk Ratio IV, Random, 95% CI
**6.3.1 DHA alone**						
Collins et al,[25] 2017	0.1222	0.0523	592	613	34.4%	1.13 [1.02, 1.25]
Manley et al,[3] 2011	-0.2877	0.14	145	149	23.5%	0.75 [0.57, 0.99]
Marc et al,[27] 2020	0.3075	0.1224	252	229	25.7%	1.36 [1.07, 1.73]
**Subtotal (95% CI)**			**989**	**991**	**83.6%**	**1.06 [0.81, 1.39]**
Heterogeneity: Tau² = 0.05; Chi² = 10.71, df = 2 (P = 0.005); I² = 81%						
Test for overall effect: Z = 0.44 (P = 0.66)						
**6.3.2 DHA + AA**						
Hellström et al,[26] 2021	0.1044	0.2069	101	105	16.4%	1.11 [0.74, 1.67]
**Subtotal (95% CI)**			**101**	**105**	**16.4%**	**1.11 [0.74, 1.67]**
Heterogeneity: Not applicable						
Test for overall effect: Z = 0.50 (P = 0.61)						
**Total (95% CI)**			**1090**	**1096**	**100.0%**	**1.07 [0.86, 1.34]**
Heterogeneity: Tau² = 0.03; Chi² = 10.71, df = 3 (P = 0.01); I² = 72%						
Test for overall effect: Z = 0.63 (P = 0.53)						
Test for subgroup differences: Chi² = 0.03, df = 1 (P = 0.86), I² = 0%						

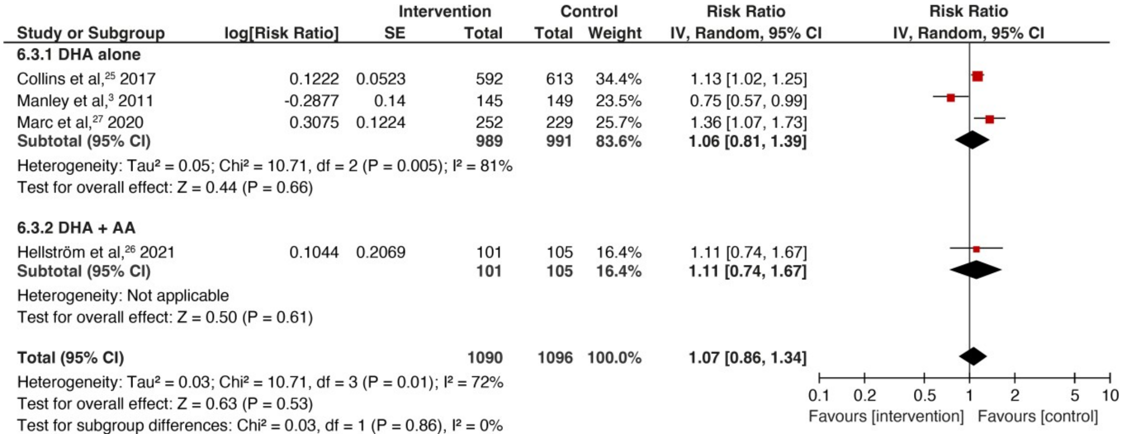

AA: Arachidonic acid; BPD: Bronchopulmonary dysplasia; CI: Confidence interval; DHA: Docosahexaenoic acid; IV: Inverse variance; PMA: Postmenstrual age; SE: Standard error.

[a]Physiological BPD based on oximetry[28] except in Hellström et al,[26] 2021 and Manley et al,[3] 2011 where BPD was with no oximetry assessment.

© 2023 Marc I et al. *JAMA Network Open.*

eReferences

1. Abou El Fadl DK, Ahmed MA, Aly YA, Darweesh EAG, Sabri NA. Impact of Docosahexaenoic acid supplementation on proinflammatory cytokines release and the development of Necrotizing enterocolitis in preterm Neonates: A randomized controlled study: Impact of Docosahexaenoic acid supplementation on proinflammatory cytokines. *Saudi Pharmaceutical Journal.* 2021.
2. Atwell K, Collins CT, Sullivan TR, et al. Respiratory hospitalisation of infants supplemented with docosahexaenoic acid as preterm neonates. *J Paediatr Child Health.* 2013;49(1):E17-22.
3. Manley BJ, Makrides M, Collins CT, et al. High-dose docosahexaenoic acid supplementation of preterm infants: respiratory and allergy outcomes. *Pediatrics.* 2011;128(1):e71-77.
4. Baack ML, Puumala SE, Messier SE, Pritchett DK, Harris WS. Daily Enteral DHA Supplementation Alleviates Deficiency in Premature Infants. *Lipids.* 2016;51(4):423-433.
5. Bernabe-García M, Villegas-Silva R, Villavicencio-Torres A, et al. Enteral Docosahexaenoic Acid and Retinopathy of Prematurity: A Randomized Clinical Trial. *JPEN J Parenter Enteral Nutr.* 2019;43(7):874-882.
6. Bernabe-García M, Calder PC, Villegas-Silva R, et al. Efficacy of Docosahexaenoic Acid for the Prevention of Necrotizing Enterocolitis in Preterm Infants: A Randomized Clinical Trial. *Nutrients.* 2021;13(2).
7. Carlson SE, Werkman SH, Tolley EA. Effect of long-chain n-3 fatty acid supplementation on visual acuity and growth of preterm infants with and without bronchopulmonary dysplasia. *Am J Clin Nutr.* 1996;63(5):687-697.
8. Carlson SE, Montalto MB, Ponder DL, Werkman SH, Korones SB. Lower incidence of necrotizing enterocolitis in infants fed a preterm formula with egg phospholipids. *Pediatr Res.* 1998;44(4):491-498.
9. Clandinin MT, Van Aerde JE, Parrott A, Field CJ, Euler AR, Lien EL. Assessment of the efficacious dose of arachidonic and docosahexaenoic acids in preterm infant formulas: fatty acid composition of erythrocyte membrane lipids. *Pediatr Res.* 1997;42(6):819-825.
10. Clandinin MT, Van Aerde JE, Merkel KL, et al. Growth and development of preterm infants fed infant formulas containing docosahexaenoic acid and arachidonic acid. *J Pediatr.* 2005;146(4):461-468.
11. Fewtrell MS, Morley R, Abbott RA, et al. Double-blind, randomized trial of long-chain polyunsaturated fatty acid supplementation in formula fed to preterm infants. *Pediatrics.* 2002;110(1 Pt 1):73-82.
12. Frost BL, Patel AL, Robinson DT, Berseth CL, Cooper T, Caplan M. Randomized Controlled Trial of Early Docosahexaenoic Acid and Arachidonic Acid Enteral Supplementation in Very Low Birth Weight Infants. *J Pediatr.* 2021;232:23-30 e21.
13. Khalesi N, Bordbar A, Khosravi N, Kabirian M, Karimi A. The Efficacy of Omega-3 Supplement on Prevention of Retinopathy of Prematurity in Premature Infants: A Randomized Double-blinded Controlled trial. *Curr Pharm Des.* 2018;24(17):1845-1848.
14. Makrides M, Gibson RA, McPhee AJ, et al. Neurodevelopmental outcomes of preterm infants fed high-dose docosahexaenoic acid: a randomized controlled trial. *JAMA.* 2009;301(2):175-182.
15. Manley BJ, Makrides M, Collins CT, Gibson RA, Ryan P, Davis PG. Allergy and respiratory outcomes from the DINO (DHA for the improvement of neurodevelopmental outcome in preterm) trial. *J Paediatr Child Health.* 2010;46:39-40.
16. Moltu SJ, Blakstad EW, Strømmen K, et al. Enhanced feeding and diminished postnatal growth failure in very-low-birth-weight infants. *J Pediatr Gastroenterol Nutr.* 2014;58(3):344-351.
17. Osman WOA, Samie Awad HAE, El-Deen OGB, Mahmoud NMK, Mohamed SMH. Effect of DHA on pro inflammatory cytokines including Platelets Activating Factor (PAF) and it's role in prevention of necrotizing enterocolitis in preterm/very low birth weight neonates. *Current Pediatric Research.* 2021;25(10).
18. Picaud JC, Reynolds P, Clarke P, et al. A novel human milk fortifier supports adequate growth in very low birth weight infants: A randomised controlled trial. *J Pediatr Gastroenterol Nutr.* 2022;74(2):930-931.
19. Rigo J, Hascoët JM, Billeaud C, et al. Growth and Nutritional Biomarkers of Preterm Infants Fed a New Powdered Human Milk Fortifier: A Randomized Trial. *J Pediatr Gastroenterol Nutr.* 2017;65(4):e83-e93.
20. Valentine CJ, Dingess KA, Kleiman J, Morrow AL, Rogers LK. A Randomized Trial of Maternal Docosahexaenoic Acid Supplementation to Reduce Inflammation in Extremely Preterm Infants. *J Pediatr Gastroenterol Nutr.* 2019;69(3):388-392.
21. Vanderhoof J, Gross S, Hegyi F, Multicenter Study G. A multicenter long-term safety and efficacy trial of preterm formula supplemented with long-chain polyunsaturated fatty acids. *J Pediatr Gastroenterol Nutr.* 2000;31(2):121-127.

© 2023 Marc I et al. *JAMA Network Open.*

22. Strømmen K, Lyche JL, Blakstad EW, et al. Increased levels of phthalates in very low birth weight infants with septicemia and bronchopulmonary dysplasia. *Environ Int.* 2016;89-90:228-234.
23. Strømmen K, Haag A, Moltu SJ, et al. Enhanced nutrient supply to very low birth weight infants is associated with higher blood amino acid concentrations and improved growth. *Clin Nutr ESPEN.* 2017;18:16-22.
24. Westerberg AC, Schei R, Henriksen C, et al. Attention among very low birth weight infants following early supplementation with docosahexaenoic and arachidonic acid. *Acta Paediatr.* 2011;100(1):47-52.
25. Collins CT, Makrides M, McPhee AJ, et al. Docosahexaenoic Acid and Bronchopulmonary Dysplasia in Preterm Infants. *New England Journal of Medicine.* 2017;376(13):1245-1255.
26. Hellstrom A, Nilsson AK, Wackernagel D, et al. Effect of Enteral Lipid Supplement on Severe Retinopathy of Prematurity: a Randomized Clinical Trial. *JAMA Pediatr.* 2021:E1-E9.
27. Marc I, Piedboeuf B, Lacaze-Masmonteil T, et al. Effect of Maternal Docosahexaenoic Acid Supplementation on Bronchopulmonary Dysplasia-Free Survival in Breastfed Preterm Infants: A Randomized Clinical Trial. *JAMA.* 2020;324(2):157-167.
28. Walsh MC, Wilson-Costello D, Zadell A, Newman N, Fanaroff A. Safety, reliability, and validity of a physiologic definition of bronchopulmonary dysplasia. *J Perinatol.* 2003;23(6):451-456.
29. Jobe AH, Bancalari E. Bronchopulmonary dysplasia. *Am J Respir Crit Care Med.* 2001;163(7):1723-1729.

© 2023 Marc I et al. *JAMA Network Open.*
