# Supplementary material for: Association Between Enteral Supplementation With High-Dose Docosahexaenoic Acid and Risk of Bronchopulmonary Dysplasia in Preterm Infants: A Systematic Review and Meta-analysis
Source: JAMA Netw Open. 2023 Mar 21;6(3):e233934. doi: 10.1001/jamanetworkopen.2023.3934 (PMC10031388; doi:10.1001/jamanetworkopen.2023.3934)
Supplement: Supplement 2. — Data Sharing Statement [file jamanetwopen-e233934-s002.pdf]

Data Sharing Statement

Marc. Association Between Enteral Supplementation With High-Dose Docosahexaenoic Acid and Risk of Bronchopulmonary Dysplasia in Preterm Infants. *JAMA Netw Open*. Published March 21, 2023. doi:10.1001/jamanetworkopen.2023.3934

Data
Data available: No

Additional Information
Explanation for why data not available: This systematic review and meta-analysis did not generate original data. Data used remain available at the cited references.
